# Computational modeling of decision-making in substance abusers: testing Bechara’s hypotheses

**DOI:** 10.3389/fpsyg.2024.1281082

**Published:** 2024-05-31

**Authors:** Laurent Avila Chauvet, Diana Mejía Cruz

**Affiliations:** Psychology Department, Sonora Institute of Technology, Obregon City, Sonora, Mexico

**Keywords:** computational modeling, linear operator model, decision-making, agents, sensitivity to losses, sensitivity to gains, substance abuse

## Abstract

One of the cognitive abilities most affected by substance abuse is decision-making. Behavioral tasks such as the Iowa Gambling Task (IGT) provide a means to measure the learning process involved in decision-making. To comprehend this process, three hypotheses have emerged: (1) participants prioritize gains over losses, (2) they exhibit insensitivity to losses, and (3) the capacity of operational storage or working memory comes into play. A dynamic model was developed to examine these hypotheses, simulating sensitivity to gains and losses. The Linear Operator model served as the learning rule, wherein net gains depend on the ratio of gains to losses, weighted by the sensitivity to both. The study further proposes a comparison between the performance of simulated agents and that of substance abusers (*n* = 20) and control adults (*n* = 20). The findings indicate that as the memory factor increases, along with high sensitivity to losses and low sensitivity to gains, agents prefer advantageous alternatives, particularly those with a lower frequency of punishments. Conversely, when sensitivity to gains increases and the memory factor decreases, agents prefer disadvantageous alternatives, especially those that result in larger losses. Human participants confirmed the agents’ performance, particularly when contrasting optimal and sub-optimal outcomes. In conclusion, we emphasize the importance of evaluating the parameters of the linear operator model across diverse clinical and community samples.

## 1 Introduction

Substance abuse represents serious health problems and has shown a high correlation with family issues and criminal behavior (UNODC, World Drug Report, 2023). It has been reported that decision-making is one of the cognitive functions most affected (Kluwe-Schiavon et al., 2020b). In temporal discounting tasks, individuals with substance abuse, particularly related to cocaine, heroin, alcohol, methamphetamine, and tobacco, tend to choose immediate, smaller rewards over larger, delayed rewards; they exhibit a more pronounced discounting rate for delayed gratification (Story et al., 2016; Gowin et al., 2018). Based on these facts, it is reasonable to infer that there is a sensitivity to gain, just like reinforcement sensitivity theory (Corr, 2004).

The Iowa Gambling Task (IGT) is a behavioral task designed to assess the decision-making process in situations of uncertainty and risk. In this task, participants are asked to choose among four alternatives over a series of trials, which differ in their gains, losses, and probability of losses. Initially, participants make somewhat random choices, but once they become familiar with the alternatives, participants tend to choose long-term advantageous alternatives or long-term disadvantageous alternatives based on the properties of the alternatives (Bechara et al., 1994).

This task has been used to assess decision-making in participants with ventromedial damage to the prefrontal cortex and individuals with a history of substance use disorder (Bechara et al., 1994; Barry and Petry, 2008; Baitz et al., 2021). In general terms, healthy participants or those in control groups have shown better performance or a preference for long-term advantageous alternatives (Bechara et al., 1994; Barry and Petry, 2008; Kluwe-Schiavon et al., 2020b; Baitz et al., 2021). On the other hand, individuals with substance use disorders tend to choose cards that offer higher short-term gains but also larger penalties, which could imply a reduced sensitivity to punishments. It has been reported that anticipatory responses, measured through skin conductance responses in participants with prefrontal lobe damage, tend to be lower than those of control participants (Bechara et al., 1997). Understanding the elements of this process can help with an early diagnosis and treatment for substance disorders, as well as assessing the development of decision-making processes during adolescence and the decline in decision-making after the age of 60 (Beitz et al., 2014).

Several cognitive and computational models have been used to simulate and understand decision-making in the IGT. Notably, Expectancy Valence (EV) and Prospective Valence Learning (PVL) capture distinct psychological processes, including expectancy, motivation, memory, and response consistency (Horstmann et al., 2012; Steingroever et al., 2013). A hybrid version of these models is the PVL-Delta, which employs the Linear Operator or Delta learning rule and is more effective to simulate decision-making in the IGT (Steingroever et al., 2014). Moreover, other Bayesian models have attempted to simulate decision-making in the IGT. These models consider gain sensitivity, loss sensitivity, and payout memory, allowing for a more parsimonious understanding of decision-making and its clinical implications (Wetzels et al., 2010).

Concerning individuals with substance abuse, a recent study uses the computational model-MAIDEN-IGT-to evaluate the decision-making process in the IGT (Iglesias et al., 2012; Serrano et al., 2022). Unlike the previously described models, this model aims to assess the weight of connections between components (i.e., Remaining trials, Accumulated money, Last outcome positivity, Last outcome negativity, among others) based on adjusting the outcomes obtained and the behavior of real participants. This model accounted for over 80% of the participant’s behavior and substantiated that methamphetamine users showed a reduced perception of potential and associated risk losses (Serrano et al., 2022).

Despite the numerous cognitive and computational models used to assess decision-making processes, few models have directly compared the performance of healthy controls and substance abusers or directly tested the hypotheses suggested in the IGT article (Bechara et al., 1997). The MAIDEN-IGT model ignores the initial 20 trials of the IGT (Serrano et al., 2022), which allows for observing the decision-making transition between uncertain and risk situations. Conversely, the PVL-Delta model omits healthy participants who exhibit random choice behaviors (Steingroever et al., 2013), which can be important to understanding the decision-making process.

To fill these gaps, we propose a simpler agent model that integrates “matching” as the decision rule and the linear operator as the learning rule, which in combination has shown better adaptation to environmental changes in other Agent-Based Models (ABM) (Beauchamp, 2000). Additionally, to test Bechara’s hypotheses in the decision-making process, we incorporate two parameters into net gains: sensitivity to losses and sensitivity to gains. The three hypotheses proposed by Bechara et al. (1997) to be examined are: (A) Participants display heightened sensitivity to rewards. (B) Participants show insensitivity to punishment. (C) Participants exhibit a lack of responsiveness to future consequences or problems or working memory. Furthermore, we compare the performance of simulated agents with that of substance abusers and control adults by manipulating the memory factor and sensitivity to losses and gains. Through this simulation, we can contrast the decision-making process alongside the performance of the IGT among the groups.

### 1.1 Linear operator model

**Figure 1:**
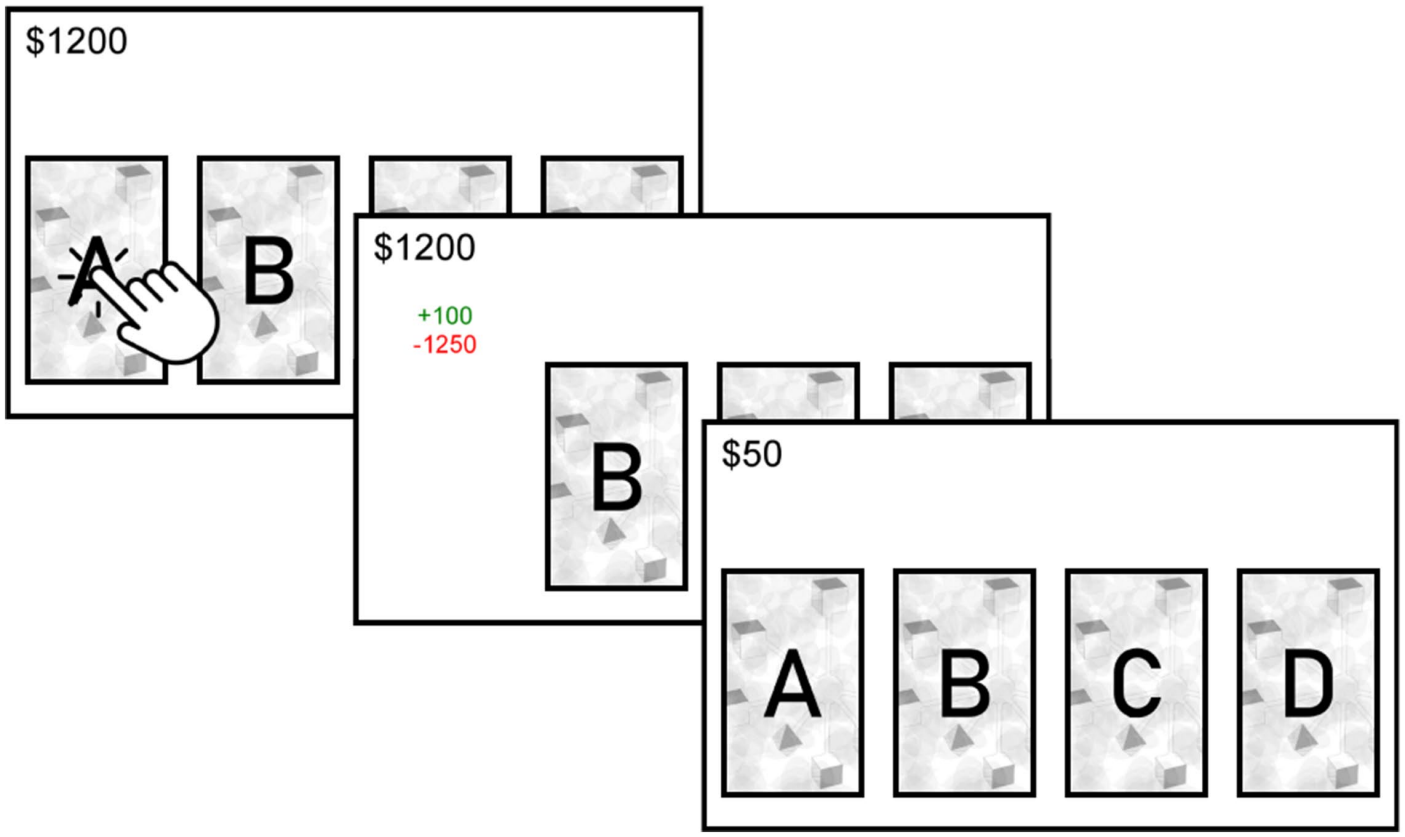
Screenshot of the Iowa Gambling Task.

Agent-Based Models (ABMs) abstract some of the relationships and components of real systems. In this model, an agent is simulated to make choices over 100 trials among the four available alternatives in the IGT (i.e., Figure 1). The alternatives differ in gains (*G*), losses (*L*), and probability of losses (*P*). Two of the alternatives are advantageous in the long term (*A: G = 100, L = −1,250, p = 0.1; B: G = 100, L = −250, p = 0.5*), while two of the alternatives are disadvantageous in the long term (*C: G = 50, L = −50, p = 0.5; D: G = 50, L = −250, p = 0.1*). To simplify computations, the values of gains and losses were normalized by using the maximum possible gain value from one of the alternatives (i.e.*, G: 50/100 = 0.5; L: −1250/100 = −12.5*).

When the agent chose one of the alternatives, the net gain (*G^n^*) was calculated by subtracting the losses from the gains. To test the hypotheses initially proposed by Bechara et al. (1997), gains were weighted according to their sensitivity to gains (Gs), while losses were weighted according to their sensitivity to losses (Sp). The sensitivity parameter values ranged from 0.1 to 0.9 (Equation 1). As the sensitivity parameter value decreased, the agent’s perception of loss or gain also decreased, capturing the contrast between losses and gains. For example, let us consider two agents with different sensitivity levels: one has a high sensitivity to losses (0.9) and a low sensitivity to gains (0.1), while the other has a low sensitivity to losses (0.1) and a high sensitivity to gains (0.9). If option B in a trial offers a gain of $100 and a loss of $250, the net value for the first agent would be −215, decreasing the value of option B, while for the second agent it would be +65, increasing the value of option B.


(1) $$G^{n}=G\left(G^{s}\right)-L\left(L^{s}\right)$$

For the agents, each alternative was associated with a value (*V^A^, V^B^,V^C^,V^D^*). At the start of each simulation, each alternative’s value was the same (*V^x^* = 0.25). This value changed according to the Linear Operator learning rule, which has shown greater flexibility compared to the perfect memory and relative payoff sum learning rules (Beauchamp, 2000). After each choice, the alternative’s value was updated based on the memory factor (*M*) and its previous value (Equation 2). When the memory factor is higher, the agent will tend to give more weight to the value of the alternative in the past time step (*MV^xt-1^*), while when the memory factor is lower, the agent will give more weight to the present net gains (*1-M*) *G^n^*.


(2) $$V^{xt}=MV^{xt-1}+\left(1-M\right)\;G^{n}$$

The probability of choosing an alternative (*P^xt + 1^*) will depend on the value associated with each alternative and the Matching decision rule. This simple rule, as used in other ABMs (Beauchamp, 2000), allows agents to explore alternatives even when their values are relatively low, without the need for additional parameters such as the temperature used in the Softmax decision rule. The value of each alternative is divided by the sum of the values of all alternatives (Equation 3). Each alternative obtains a value range between 0 and 1, which adds up to 1 (i.e., *P^A^ = 0.1; P^B^ = 0.3; P^C^ = 0.4; P^D^ = 0.2*). Subsequently, the alternatives are arranged in ranges according to their probability (*P^A^ < P ^A + B^ < P ^A + B + C^ < P ^A + B + C + D^*), and the agent chooses one of the alternatives based on a random number between 0 and 1. If the value of the alternative increases, its probability of being chosen also increases. The Supplementary material includes the MATLAB code of a version of the model using Softmax as the decision rule.


(3) $$P^{x}=V^{x}/\left(V^{A}+V^{B}+V^{C}+V^{D}\right)$$

### 1.2 Parametric variations simulation

**Figure 2:**
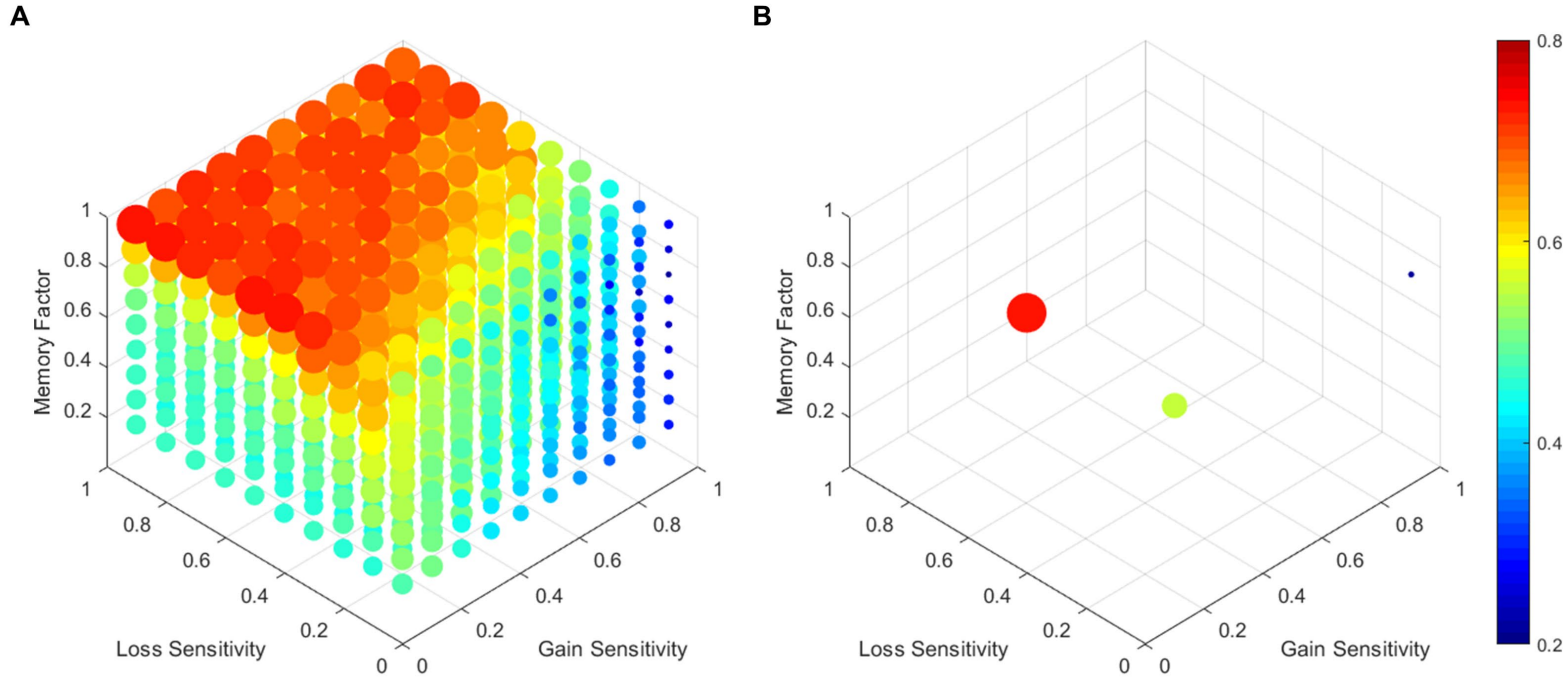
Relative preference parameter combination. Each data point represents the mean relative preference for choices of 50 agents. Points with a red hue and larger size indicate a preference for advantageous choices, while smaller blue points reflect a preference for disadvantageous choices. **(A)** displays the combination of parameter values, while **(B)** exclusively illustrates the parameter combinations that resulted in the maximum, minimum, and average relative preference.

To assess the relative preference for the advantageous choices (*(A + B)/(A + B + C + D)*), we systematically varied the gain sensitivity, the loss sensitivity, and the memory factor in increments of 0.1. A total of 900 simulations were conducted, with 50 simulations for each parameter combination. Figure 2A shows the mean relative preference for the advantageous choices across the 50 simulations for each parameter combination. Figure 2B displays the combination of parameters that resulted in a higher preference for advantageous (*M* = 0.9, *G^s^* = 0.1, *L^s^* = 0.5) and disadvantageous alternatives (*M* = 0.7, *G^s^* = 1, *L^s^* = 0.1), as well as the mean preference of all simulations (*M* = 0.6, *G^s^* = 0.3, *L^s^* = 0.2). The agents tend to choose advantageous alternatives as the memory factor and the loss sensitivity increase. The Supplementary material includes the results of each parameter combination and a version of the MATLAB 2020 code used to perform the simulations for a more detailed review.

## 2 Method

### 2.1 Participants and design

A cross-sectional design was employed, along with a snowball sampling strategy, for recruiting healthy control participants. Recruitment took place for individuals with substance abuse at a residential addiction treatment center in northern Sinaloa, Mexico. These participants were in their third week of abstinence. Each participant was assigned an ID number to ensure confidentiality. Participants accepted an informed consent form indicating their voluntary participation and that the data collected would only be used for research purposes. The protocol was approved by the Sonora Institute of Technology Institutional Review Board (ID 84).

Owing to COVID-19 restrictions, the substance abuse group consisted of 20 male participants who met the criteria for substance use disorders as established in psychiatric reports (age: M = 32.07, SD = 14.10). The healthy control group was formed with 20 participants to match the sample size of the substance abuse group (males: *n* = 16; females: *n* = 4; age: M = 23.08, SD = 12.61). Participants in the healthy group did not meet the criteria for substance use disorders according to the DSM-5 criteria (American Psychiatric Association, 2013).

### 2.2 Instruments

A demographic survey and an online version of the IGT analogous to the one used by Bechara et al. (1994) were employed (Figure 1). IGT involves four decks that vary in terms of gains, losses, and probability of losses. Among these alternatives, two were advantageous in the long term (*A: G* = 100, *L* = −1,250, *p* = 0.1; *B: G* = 100, *L* = −250, *p* = 0.5), while the other two were disadvantageous in the long term (*C: G* = 50, *L* = −50, *p* = 0.5; *D: G* = 50, *L* = −250, *p* = 0.1). Participants were instructed to choose one of the four cards and advised to try to win the maximum amount of money possible. They were also informed that:

“At the top of the chosen deck, the amount of money won will appear in green, and the amount of money lost will appear in red. In the upper left corner, the accumulated money will be displayed (i.e., $100 – $50 = $50).”

The task ended when they chose a total of 100 cards. Data collection occurred on a secure server accessible only to the primary researchers.

### 2.3 Procedure

The study was conducted over two months (July 31, 2021, to September 29, 2021). Participants voluntarily took part in the study. The procedures adhered to the Helsinki Declaration on human research participation: (1) Data confidentiality and privacy; (2) Informed consent; (3) Publication and research records; (4) Dissemination of results. Participants had the option to request additional information via the lab’s email.

All participants accessed the same website developed for this study. The link to the website was sent to healthy control participants so they could access the task on their mobile devices. In contrast, participants in the substance abuse group accessed the website through a tablet provided by researchers at the residential center. The assessments took place in the psychology offices of the drug rehab facilities, taking into consideration the precautions recommended by the Mexican Ministry of Health to prevent the spread of COVID-19. To evaluate individuals within the substance abuse group, participants had to meet specific inclusion criteria. These criteria included having no schizophrenia events or other psychotic disorders and no withdrawal symptoms, as reported by the psychiatrist in their file. Once selected to participate, the researcher read the informed consent aloud to the participant and then assigned an ID folio.

**Figure 3:**
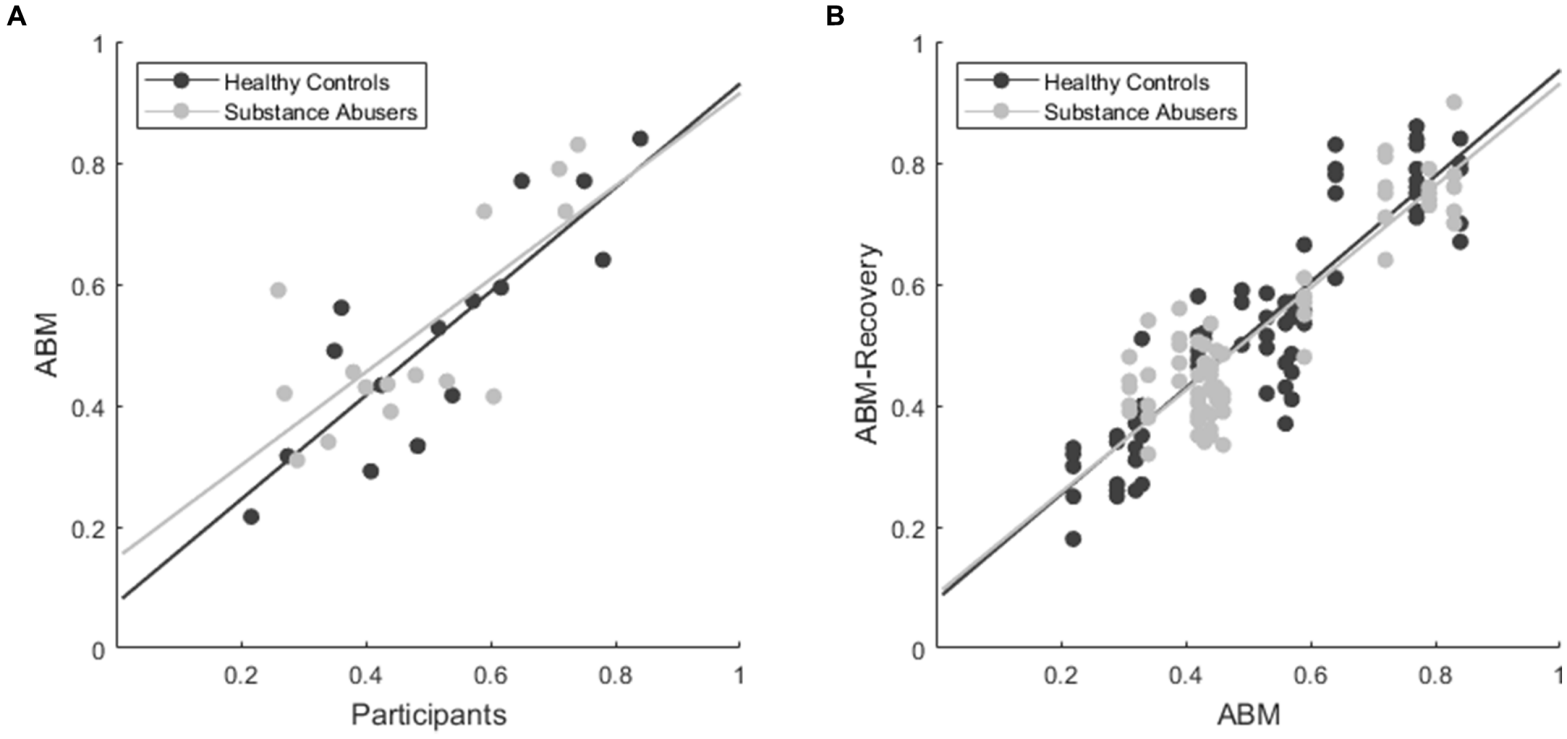
Correlation of relative preference for advantageous choices. **(A)** shows the correlation between the mean relative preference for advantageous choices in the first four blocks for both participants and agents. Meanwhile, **(B)** shows the correlation between the preference for advantageous choices of the fitted Agent-Based Model (Figure 3A) and five replicas using the recovered parameters of memory, gain sensitivity, and loss sensitivity.

MATLAB 2020 was used to conduct the simulations, assigning a single simulated agent to each participant. This resulted in 40 agents, evenly distributed between 20 agents for the healthy control group and 20 for the substance abuse group. To find the optimal values of the memory, gain sensitivity, and loss sensitivity parameters of the agents that would result in a relative preference for advantageous choices of the participants’ last block, a while loop was implemented. Before an agent was exposed to the task, its characteristics or parameter values for memory, gain sensitivity, and loss sensitivity were randomly selected. If the relative preference for advantageous choices in the fifth block of the agents deviated by more than 0.01 from the relative preference for advantageous choices in the fifth block of the participants, new random values were generated for memory, gain sensitivity, and loss sensitivity. Subsequently, the agents were exposed to the task again until the agents’ preference in the fifth block did not deviate by more than 0.01 from the relative preference for advantageous choices in the fifth block of the participants. The optimal parameter estimation was conducted in this manner because one of the aims of the was to simulate the evolution of preference for the alternatives across blocks using the same initial traits or characteristics. However, it is important to highlight that parameter recovery was also estimated by testing the Nelder–Mead method (see Supplementary material). Although the fit was better, concerns regarding the replicability of the data using new agents, the range of parameter variability, and the computational time led us to utilize the described method. For further details, please refer to the MATLAB and Python code provided in the Supplementary material. To ensure that the model could replicate similar decision-making processes with the known parameters that simulated the participants’ decision-making (Figure 3), we conducted a new simulation with 5 groups of 40 agents, using the recovered parameters of memory, gain sensitivity, and loss sensitivity.

### 2.4 Data analysis

Concerning demographic characteristics such as age, education, and income levels, along with the frequency of methamphetamine use (in grams), we compute the medians, means and standard deviations. We carry out Kolmogorov–Smirnov, Shapiro–Wilk, and Levene’s tests to determine the appropriate choice between parametric and non-parametric statistical tests.

To evaluate the mean relative preference for advantageous choices in each block, we divided the sum of the frequency of choosing alternatives *A* and *B* by the total possible choices in each block (20). Subsequently, three subgroups were formed based on the proportion of advantageous cards selected in the Iowa Gambling Task for both groups, participants, and agents. Group Q3 includes those whose proportions are in the third quartile or above it; Group Q1 encompasses those below the first quartile; and Group Q2 comprises participants with proportions between the first and third quartiles. A repeated measures ANOVA was utilized to analyze the differences in the preference for advantageous choices across Blocks, Groups, and Subgroups. Additionally, a power function (*y = ax^b^*) was fitted to the blocks of preference for advantageous choices for each group and subgroup. The parameter b of the power function indicates the rate of change in preference; values far from 0 indicate a learning process of the properties of the alternatives (Mejía et al., 2022). Moreover, in order to evaluate the relative weight of demographic characteristics on the rate of preference change, a regression tree from the sklearn library for Python was utilized.

A correlation and was employed to assess the relationship between the Mean Relative Preference for Advantageous Choices of participants and the Agent-Based Model and the relationship between the preference for advantageous choices in the simulation of the fitted Agent-Based Model and its replicas with the recovered parameters Block 5 was excluded due to its use as simulation objectives in the ABM. The statistical analyses and power analyses were performed using the JASP statistical package and the FTestAnovaPower library in Python, respectively. MATLAB 2020 was utilized for simulations and fitting power functions.

## 3 Results

A Mann–Whitney U test was used to compare the demographic variables between the Substance abusers and controls. The analysis revealed no significant statistical differences in income level (*p* = 0.356). In contrast, there were significant differences in the level of education (*p* = 0.005), age (*p* = 0.037, *rB* = 0.38), and quantity of crystal meth consumption in grams (*p* < 0.001), see Table 1.

**Table 1:** Demographic variables between the substance abusers and controls.

*N*	Healthy controls				Substance abusers		*Uw*	*p*
	20				20			
	*Mdn*	*M*	*SD*	*Mdn*	*M*	*SD*		
Age	22.5	21.95	2.21	28.5	29.25	10.56	277.5	**0.037**
Level of education (years)	13.5	13.25	3.27	9	10.15	4.42	97.50	**0.005**
Monthly income	5,100	$6245.00	$5452.61	1,250	$8,210.4	$14,504.31	166.0	0.356
Quantity of crystal meth (grams) per occasion	0	0.20	0.41	1.5	1.90	1.29	370.0	**< 0.001**
Abstinence period (days)				75.5	84.8	65.75		

The bold *p*-values indicate statistically significant differences using a Mann–Whitney U test.

**Figure 4:**
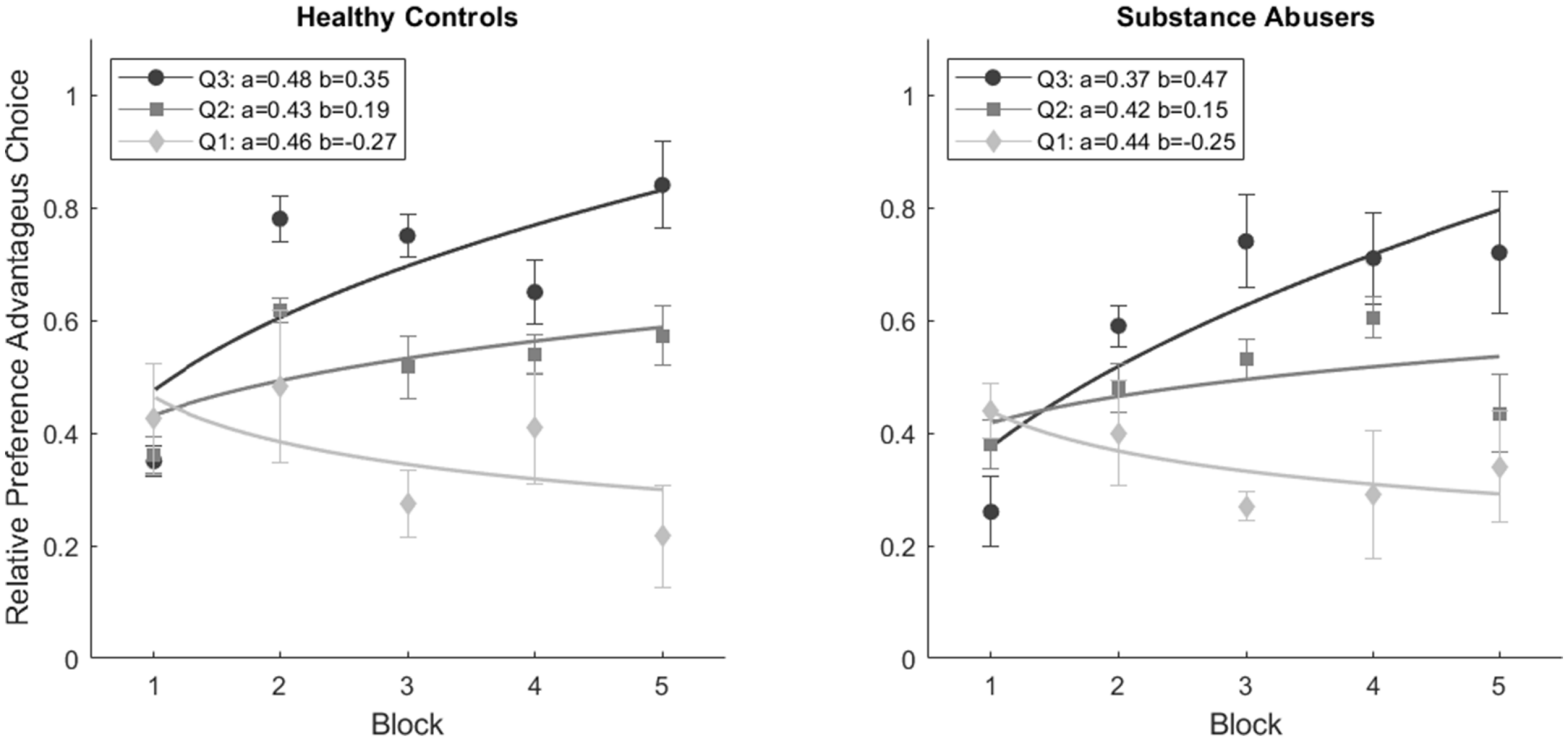
Mean relative preference for advantageous choices for groups, subgroups, and blocks. The graphical representation of fitting a power function, which indicates the rate of change reflecting the preference for advantageous alternatives over time.

Figure 4 displays the mean relative preference for advantageous choices for each block of 20 cards for the healthy control and substance abuse groups. Each group was divided based on their relative preference for advantageous choices. As expected, the subgroups belonging to the Q3 exhibited a positive rate of change in their relative preference for advantageous choices (Healthy Control: 0.35, Substance Abusers: 0.47), in contrast to the subgroups belonging to the Q1 (Healthy Control: −0.27, Substance Abusers: −0.25). For the Q2 subgroups, the rate of change remained more stable compared to the other two subgroups (Healthy Control: 0.19, Substance Abusers: −0.15). The parameter *b* of the power function indicates the shift in preference. The parameter ‘b’ of the power function indicates the change in preference. According to the regression tree, age holds the highest relative weight (*Rw* = 0.48) in predicting the rate of change, followed by the quantity of crystal (*Rw* = 0.30), income (*Rw* = 0.16), and education (*Rw* = 0.04). As age decreases, the parameter ‘b’ tends to be greater than 0.

A repeated measures ANOVA revealed significant statistical differences between blocks [*F*(4, 38) = 9.244, *p* < 0.001, ω^2^ = 0.132] and a Block*Subgroup interaction [*F*(8, 38) = 7.790, *p* < 0.001, ω^2^ = 0.132]. No significant differences were observed in Block*Group interaction [*F*(4, 38) = 1.372, *p* = 0.247, ω^2^ = 0.007] and Block*Group*Subgroup interaction [*F*(4, 38) = 1.194, *p* = 0.307, ω^2^ = 0.004]. The *post hoc* analyses showed no significant differences between Healthy Controls and Substance Abusers (*p* = 0.229). However, notable differences were observed among subgroups, particularly between Q1 vs. Q2 (*p* < 0.001), Q1 vs. Q3 (*p* < 0.001), and Q2 vs. Q3 (*p* = 0.001). Significant distinctions emerged between Block 1 and the remaining blocks (*p* < 0.001). Results suggest that agents with a lower relative preference for advantageous choices tend to weigh the gains more, even if they are not advantageous in the long run.

**Figure 5:**
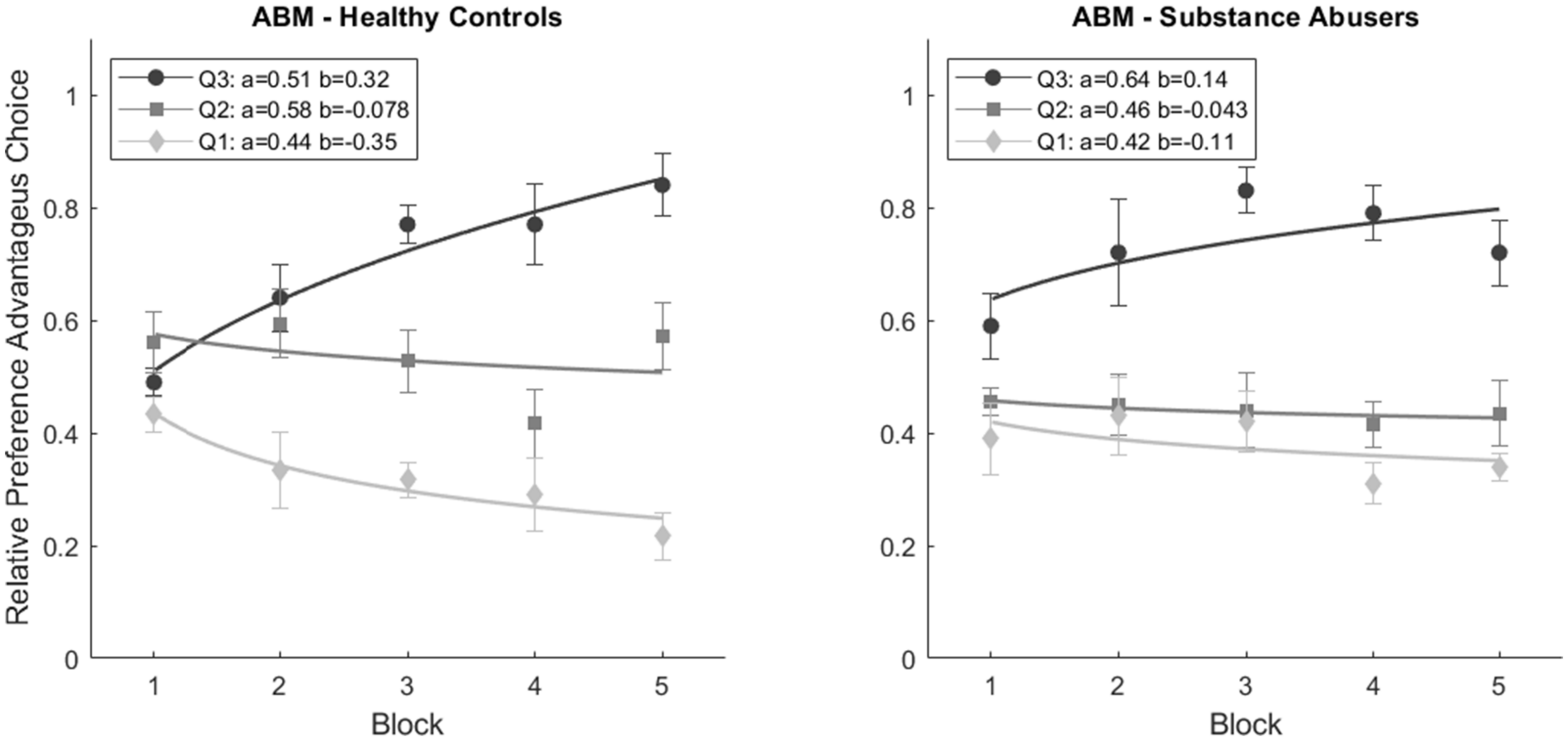
Mean relative preference for advantageous choices for ABM groups, subgroups, and blocks. The graphical representation of fitting a power function, which indicates the rate of change reflecting the preference for advantageous alternatives over time.

**Figure 6:**
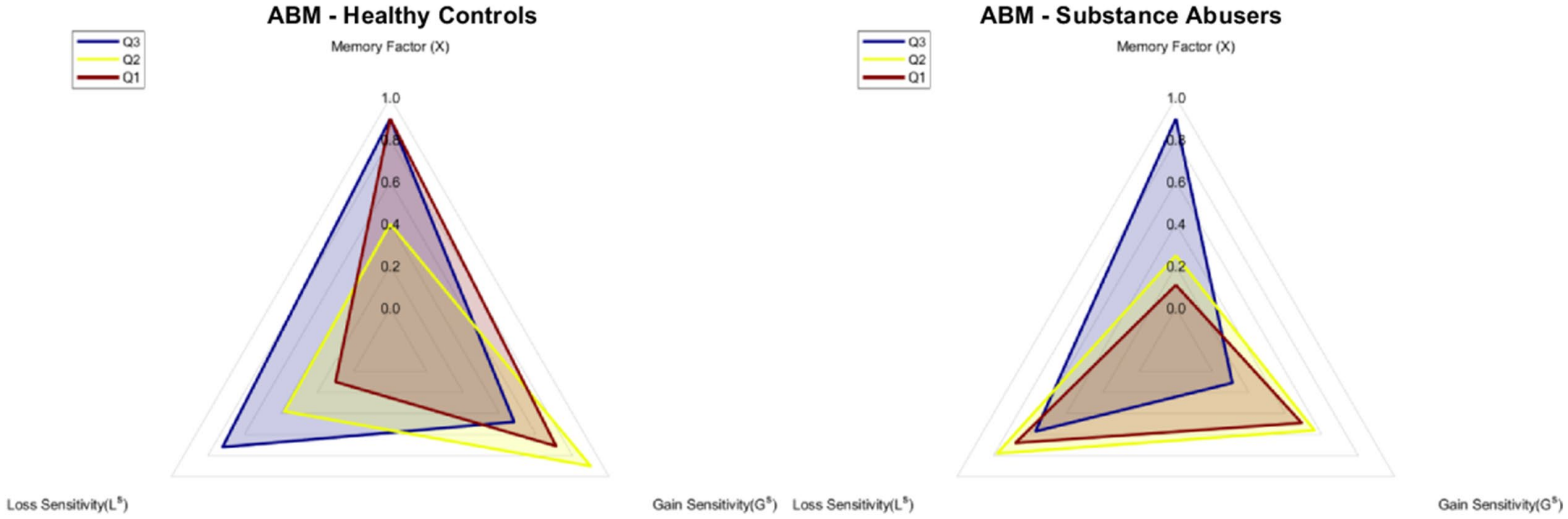
ABM simulations estimated parameter values.

Figure 5 displays the mean relative preference for advantageous choices for each block of 20 trials for the 40 agents that simulated each one of the 40 participants in each group (the parameters used for each subgroup are described in Figure 6). Similarly, to participants, the agents from the Q3 subgroups exhibited a positive rate of change in their relative preference for advantageous choices (ABM-Healthy Control: 0.32, ABM-Substance Abusers: 0.14), in contrast to the subgroups from Q1 (ABM-Healthy Control: −0.35, ABM-Substance Abusers: −0.11). On the other hand, the parameter b of the power function remained more stable for the Q2 subgroups compared to the other two subgroups (ABM-Healthy Control: 0.07, ABM-Substance Abusers: 0.043). A repeated measures ANOVA revealed significant statistical differences a Block*Subgroup interaction [*F*(8, 38) = 3.267, *p* = 0.002, ω^2^ = 0.055]. The *post hoc* analyses showed differences between ABM-Healthy Controls and ABM-Substance Abusers (*p* = 0.006), Q1 vs. Q2 (*p* < 0.001), Q1 vs. Q3 (*p* < 0.001), and Q2 vs. Q3 (*p* = 0.001).

Figure 6 shows the estimated values of gain sensitivity, loss sensitivity, and memory factor used for the predictions of Figure 5. The parameters for the agents belonging to each of the groups were the same for every agent. It is important to highlight that, despite having the same parameters per group, each agent can vary their preference throughout the blocks, as shown in the dispersion bars in Figure 5. The memory factor of the Q3 (*M* = 0.90) and Q1 (*M* = 0.90) subgroups in the ABM-Healthy Control group was higher compared to the Q2 subgroup (*M* = 0.40). Results suggest that weighing past value is relevant to changing the preference. For the ABM-Substance Abusers group, the memory factor of the Q2 (*M* = 0.40) and Q1 (*M* = 0.11) subgroups was lower than the value of the memory factor of the Q3 (*M* = 0.90) subgroup. The agents of the ABM-Substance Abusers group weigh more the present consequences. Regarding Gain Sensitivity, agents from the Q2 (ABM-Healthy Control: *G^S^* = 0.90, ABM-Substance Abusers: *G^S^* = 0.56) and Q1 (ABM-Healthy Control: *G^S^* = 0.71, ABM-Substance Abusers: *G^S^* = 0.49) subgroups tend to be more sensitive to rewards than the agents in the Q3 subgroup (ABM-Healthy Control: *G^S^* = 0.48, ABM-Substance Abusers: *G^S^* = 0.11). Results suggest that agents with a lower relative preference for advantageous choices tend to weigh more the gains, even if they are not optimal in the long run. Finally, for the ABM-Healthy Control group, the loss sensitivity was higher in the Q3 (*L^S^* = 0.72) and Q2 (*L^S^* = 0.38) subgroups compared to the Q1 subgroup (*L^S^* = 0.10). Regarding the ABM-Substance Abusers group, the loss sensitivity remained within similar ranges (Q1: *L^S^* = 0.68; Q2: *L^S^* = 0.78; Q1: *L^S^* = 0.57). It is important to note that agents without memory issues and a lower relative preference for advantageous choices show less sensitivity to losses.

Figure 3A shows the correlation between the Mean Relative Preference for Advantageous Choices in the First Four Blocks for both Participants and Agents. Block 5 was excluded from this analysis since it was used for ABM simulation. A Pearson’s correlation between the Preference for Advantageous Choices revealed a statistically significant correlation for the Healthy control group and ABM-Healthy control group (*r* = 0.744, *p* = 0.005, RMSE = 0.114), and Substance Abusers group and ABM-Substance Abusers group (*r* = 0.688, *p* = 0.013, RMSE = 0.130). The correlation between these blocks provides insights into the decision-making process throughout the task. On the other hand, the Figure 3B shows the correlation between the preference for advantageous choices of the fitted Agent-Based Model (Figure 3A) and five replicas using the recovered parameters of memory, gain sensitivity, and loss sensitivity (Figure 6). Pearson’s correlation analysis indicated a significant correlation for the Healthy Control simulations (*r* = 0.89, *p* < 0.001, RMSE = 0.078) and the Substance Abusers simulations (*r* = 0.89, *p* < 0.001, RMSE = 0.073).

## 4 Discussion

The present study explored the differences in relative preference for advantageous choices in the Iowa Gambling Task (IGT) between healthy individuals and substance abuse users. An Agent-Based Model (ABM) was also employed, using a linear operator as a learning rule and Matching as a decision rule. The purpose of implementing this simple model was to understand the underlying process in decision-making and test the initial hypotheses proposed by Bechara et al. (1997). These objectives were achieved by manipulating the memory factor and gaining and losing sensitivity.

Concerning relative preference for advantageous choices, it was observed that in the healthy control group and the substance abuse group, there were individuals who tended to make advantageous decisions in the long term and individuals who tended to make disadvantageous decisions in the long term. Although participants with comparable preferences were identified in both groups, it is plausible that the underlying decision-making processes diverge (Wetzels et al., 2010). The agent-based model that simulated each participant revealed that the memory factor of individuals in the substance abuser group was lower compared to healthy controls. This outcome suggests that substance abusers may have memory-related issues, particularly those who tend to choose disadvantageous alternatives. However, to confirm this hypothesis, evaluating the participants’ memory factor using behavioral tasks such as the keep track, letter memory or N-Back tasks would be crucial. Bechara’s “C” Hypothesis (Bechara et al., 1997) suggests that poor decision-makers may exhibit a lack of responsiveness to future consequences, as observed in the extensive literature on delay discounting tasks (Kluwe-Schiavon et al., 2020a; Mejía et al., 2022; Acuff et al., 2023). In other studies, it has been shown that substance abusers tend to choose cards that offer short-term gains but also entail larger penalties (Bechara et al., 1994; Story et al., 2016; Gowin et al., 2018; Kluwe-Schiavon et al., 2020b). Similarly, it has been observed that drugs affect brain regions associated with working memory (Verdejo-García et al., 2010; Kluwe-Schiavon et al., 2020b).

The simulation of participants in this study also provides interesting insights about the participants’ decision-making process. The results, particularly in the healthy control group, indicate that good decision-makers exhibit a robust memory factor, a moderate gain sensitivity, and a strong loss sensitivity. These findings encourage further exploration of Bechara’s C and B hypotheses (Baitz et al., 2021), which are associated with the memory factor and participants’ insensitivity to losses. Previous studies have shown that individuals with ventromedial damage in the prefrontal lobe exhibit diminished anticipatory responses (Skin Conductance Response) to disadvantageous alternatives (Bechara et al., 1997). In the case of the substance abusers’ group, it was observed that the relative preference for disadvantageous alternatives tended to fluctuate significantly. Specifically, for groups Q1 and Q2, this preference showed significant changes in the last blocks. As a result, the model had less success in simulating this group, as agents’ preference for an alternative was influenced by net gains and the memory factor. This result could be attributed to the fact that drugs affect several executive functions. The memory factor tended to be low for participants and agents who exhibited a lower rate of change or a value of ‘*b*’ closer to zero. This finding implies that to make better decisions, it is crucial to learn from past experiences (Mejía et al., 2022). The learning factor captures how much the value of alternatives in the past is weighted.

Regarding the developed agent model, it employs very simple decision and learning rules to simulate participants’ decision-making process. Despite the method used to estimate the parameters not being as successful as the Nelder–Mead method (see Supplementary material), it showed a statistically significant correlation between the first four blocks of participants and agents, as well as the possibility of simulating similar results using these same parameters. This is important as it replicated the decision-making process, demonstrating how sensitivity to gains and losses influences the learning about alternatives. Unlike other models (Wetzels et al., 2010; Horstmann et al., 2012; Steingroever et al., 2013; Serrano et al., 2022), this one deduces the process and can infer the decision-making process of indifferent decision-makers. Parametric manipulation makes it possible to infer the decision-making process of other studies. For example, the model can suggest parameter values for the control group and experimental group of the original IGT paper (Control Group: M = 0.9, Gs = 0.6, Ls = 7, EVR Group: M = 0.7, Gs = 0.6, Ls = 0.2). However, it is important to highlight that in this simple model, the value of the alternatives depends directly on the value of the alternatives in the past and the net gains in the present. In Bechara et al.’s (1997) original publication, it is reported how healthy controls occasionally choose disadvantageous alternatives in the final blocks. In this regard, it is considered relevant to explore in the future the decision-making of the agents based on the expectations of the consequences, with the purpose of simulating the participants’ risk propensity (Palminteri and Lebreton, 2022).

A potential future analysis that can be conducted using the model is to evaluate the parameters for other studies involving parametric manipulations of the probability and magnitude of auditory signals associated with gains and losses. This analysis aims to enhance the sensitivity to reinforcement and punishments. Additionally, it involves manipulating the motivation to perform the task through monetary incentives based on the final net winnings. Subsequently, this analysis could involve categorizing these studies based on the type of patients or the characteristics of their participants.

While our findings are consistent with the existing literature, one of the biggest limitations of the study lies in the characteristics of the groups, which differed in age, gender, and educational level and small sample. In this context, age emerged as one of the factors with the greatest relative weight in predicting the estimated rate of change using the power function. As age decreases, the parameter ‘b’ tends to increase above 0. Despite the substance use disorder (SUD) group having a higher average age, the quantity of crystal was the second factor with the highest relative weight. These findings may be associated with the time of exposure to substance abuse; the length of consumption is another variable to consider in future studies. A power analysis suggests that, given the design used, the sample size should have been 120 participants, with a moderate effect size (0.5), a significance level of 0.05, and a power of 0.8. The variable ‘income’ exhibited homogeneity among the groups, while the variables age and education showed significant differences between them. It has been documented that in the case of education, substance use disorder tends to interfere with academic achievement (Rattermann, 2014). Future studies will aim to increase the sample size, homogenize the demographic characteristics of the participants, and utilize Propensity Score Matching to achieve a higher level of precision when matching substance abusers with healthy controls. However, upon subgroup analysis, both groups included participants who tended to choose advantageous and disadvantageous alternatives. Additionally, despite the relatively small sample size, the simulation of analogous agents provided valuable information on the interaction between reward sensitivity and memory. It is also important to note that this model simplifies the complexity of human behavior, and other factors, such as motivation to respond to the task, may not have been considered. Future studies will aim to integrate these factors, we recommend evaluating cognitive flexibility (i.e., reversal task), delay discounting, and working memory to enhance the model’s input and simulate preferences for optimal alternatives. This combination of factors is expected to serve as a robust predictor of decision-making and risk-related behaviors.

Considering the insights derived from the ABM approach, we suggest improvements in several treatments that face the primary challenge of modifying decision-making. This challenge is particularly significant in the context of treatments for substance use disorders, impulse control disorders, and dysexecutive syndrome in general. Treatments such as Dialectical Behavioral Therapy (Soler et al., 2016; Cavicchioli et al., 2023) and Acceptance and Commitment Therapy (Morrison et al., 2014) can be enhanced through cognitive bias modification (Cristea et al., 2016; Leung et al., 2017), aiming to improve attention to the environment for the detection of various factors: signals of reinforcement or punishment, the delay and probability of outcomes, the effort involved, and the working memory required to retain this information over an extended period of operation (Bickel et al., 2011). Additionally, skills such as mindfulness, in conjunction with cognitive bias modification, can further enhance the effectiveness of these treatments.

The development of neurocomputational models has enabled us to understand the cognitive mechanisms involved in decision-making. Current findings have revealed that models relying on reinforcement learning (RL) algorithms, and Bayesian inference, which focus on vulnerabilities related to model-free and model-based control, can explain maladaptive choices despite adverse consequences in behavioral tasks such as the Iowa Gambling Task (IGT) (Lin et al., 2019; Kato et al., 2023). In the current study, we aimed to explore reward-processing biases using a simpler agent model without introducing additional parameters. This approach is distinct from other models; however, we deem it important to conduct a model comparison with other well-established models (e.g., Expected Value [EV], Prospect Valence Learning [PVL], PVL-delta, Value Propagation Process [VPP], Outcome Representation Learning [ORL], Value Similarity Encoding [VSE], Q-Learning [QL], Procedural learning [PL]) (Gonzalez et al., 2010; Horstmann et al., 2012; Steingroever et al., 2014; Ligneul, 2019; Serrano et al., 2022) to evaluate the advantages of directly utilizing the values of the alternatives without introducing additional parameters. Our findings suggest that human participants validated the agent’s performance by contrasting optimal and sub-optimal outcomes. This underscores the significance of assessing the parameters of the linear operator model across diverse clinical and community samples.

## Data availability statement

The original contributions presented in the study are included in the article/Supplementary material, further inquiries can be directed to the corresponding author.

## Ethics statement

The Sonora Institute of Technology Institutional Review Board (ID 84) approved the protocol. The studies were conducted in accordance with the local legislation and institutional requirements. The participants provided their written informed consent to participate in this study.

## Author contributions

LA: Conceptualization, Data curation, Formal analysis, Investigation, Methodology, Software, Validation, Writing – original draft, Writing – review & editing. DM: Conceptualization, Formal analysis, Funding acquisition, Investigation, Methodology, Resources, Supervision, Validation, Writing – original draft.
