# Supplementary material for: Computational modeling of decision-making in substance abusers: testing Bechara’s hypotheses
Source: Front Psychol. 2024 May 31;15:1281082. doi: 10.3389/fpsyg.2024.1281082 (PMC11178135; doi:10.3389/fpsyg.2024.1281082)
Supplement: Supplementary file 1 [file Presentation_1.pdf]

Supplementary Material

Agent-Based Models (ABMs) abstract some of the relationships and components of real systems. MATLAB Code 1 shows the Iowa gambling task ABM using matching as the decision rule and the linear operator as the learning rule.

Code 1

Model IGTAMB

```
clear
clc
% rng(5,'threefry')
%IGT
Cards = 100;
Gain = [100 100 50 50];
Lose =  [1250 250 250 50];
PLose = [.1 .5 .1 .5];
%Agent
Number_Angent = 5;
Memory = .9;
Gain_Sensibility = .8;
Lose_Sensibility = .1;
for Agent = 1:Number_Angent

  %Start Agent
  Alternatives_Value = [.25 .25 .25 .25];
  Memory_X = Memory(1);
  Gain_Proportional = (Gain / max(Gain))*Gain_Sensibility(1);
  Lose_Proportional = (Lose / max(Gain))*Lose_Sensibility(1);
  Temp_Group = 1;
  %Trials
  for Step = 1:Cards

     %Choice
     Temp_Rand_Choice = rand;
     if Temp_Rand_Choice <= Alternatives_Value(1)
        Tempchoice = 1;
     elseif Temp_Rand_Choice <= sum(Alternatives_Value(1:2))
        Tempchoice = 2;
     elseif Temp_Rand_Choice <= sum(Alternatives_Value(1:3))
        Tempchoice = 3;
     elseif Temp_Rand_Choice <= sum(Alternatives_Value(1:4))
        Tempchoice = 4;
     end
     %IGT_Lose_Probability
     TempLose = 0;
     if rand <= PLose(Tempchoice)
        TempLose = Lose_Proportional(Tempchoice);
     end
     %Linear operator
     Alternatives_Value(Tempchoice) =
(Memory_X*Alternatives_Value(Tempchoice))+((1-Memory_X)*(Gain_Proportional(Tempchoice)-TempLose));
     Temp_Agent_Data(Step,7) = (Memory_X*Alternatives_Value(Tempchoice));
     Temp_Agent_Data(Step,8) = ((1-Memory_X)*(Gain_Proportional(Tempchoice)-TempLose));
     %Update Alternatives_Value
     Alternatives_Value(find(Alternatives_Value<0.01)) = .01;
     Alternatives_Value = Alternatives_Value / sum(Alternatives_Value);
     Temp_Agent_Data(Step,1:6) = [Agent Tempchoice Alternatives_Value];
```

```
  end
  Data(Agent,1:2) = [Agent Temp_Group];
  for i = 1:4
     Data(Agent,i+2) = numel(find(Temp_Agent_Data(:,2)==i)) / Cards;
  end
  Block = [20:20:Cards];
  for i = 1:length(Block)
     Data(Agent,i+6) = numel(find(Temp_Agent_Data(Block(i)-(Block(1)-1):Block(i),2) == 3 |
Temp_Agent_Data(Block(i)-(Block(1)-1):Block(i),2) == 4)) / 20;
  end
  Data(Agent,[12 13 14 15]) = [numel(find(Temp_Agent_Data(:,2)==1)) numel(find(Temp_Agent_Data(:,2)==2))...
                        numel(find(Temp_Agent_Data(:,2)==3)) numel(find(Temp_Agent_Data(:,2)==4))]/Cards;
end
figure(1)
set(gcf,'position',[100 100 1000 400])
subplot(1,2,1)
hold on
Color = [.25 .25 .25];
Data_ = Data(:,7:11);
plot(1:5,nanmean(Data_), '-o','Color',Color,'MarkerFaceColor',Color,'MarkerFaceColor',Color,'LineWidth',1.5,'MarkerSize',6)
errorbar(1:5,nanmean(Data_),std(Data_)/sqrt(length(Data_)),
'.','Color',Color,'MarkerFaceColor',Color,'MarkerFaceColor',Color,'LineWidth',.5,'MarkerSize',6)
set(gca,'xlim',[.5 5.5])
set(gca,'xtick', [1:1:5])
set(gca,'ylim',[0 1])
set(gca,'ytick', [0:.2:1])
ylabel('Relative Preference Advantageus Choice','FontSize',12)
xlabel('Block','FontSize',12)
subplot(1,2,2)
hold on
Color = [.25 .25 .25];
Data_ = Data(:,12:15);
bar(1:4,nanmean(Data_),.70,'FaceColor',Color,'EdgeColor',Color,'LineWidth',1.5)
errorbar(1:4,nanmean(Data_),std(Data_)/sqrt(length(Data_)),
'.','Color',Color,'MarkerFaceColor',Color,'MarkerFaceColor',Color,'LineWidth',.5,'MarkerSize',6)
set(gca,'xlim',[.5 4.5])
set(gca,'xtick', [1:1:4])
set(gca,'xticklabel', {'A','B','C','D'})
set(gca,'ylim',[0 1])
set(gca,'ytick', [0:.2:1])
ylabel('Relative Preference','FontSize',12)
xlabel('Block','FontSize',12)
```

MATLAB Code 2 shows the Iowa gambling task ABM using softmax as the decision rule and the linear operator as the learning rule. It should be noted that this version of the model was not utilized for the simulations described in the manuscript. The softmax function introduces a temperature parameter for decision-making.

Code 2

Model IGTAMBsoftmax

```
clear
clc
rng(5,'threefry')
%IGT
Cards = 100;
Gain = [100 100 50 50];
```

```
Lose =  [1250 250 250 50];
PLose = [.1 .5 .1 .5];
%Agent
Number_Angent = 5;
Memory = .9;
Gain_Sensibility = .8;
Lose_Sensibility = .1;
Temperature = .25;
for Agent = 1:Number_Angent

  %Start Agent
  Alternatives_Value = [.25 .25 .25 .25];
  Memory_X = Memory(1);
  Gain_Proportional = (Gain / max(Gain))*Gain_Sensibility(1);
  Lose_Proportional = (Lose / max(Gain))*Lose_Sensibility(1);
  Temp_Group = 1;
  %Trials
  for Step = 1:Cards

     %Choice
     Temp_Rand_Choice = rand;
     if Temp_Rand_Choice <= Alternatives_Value(1)
        Tempchoice = 1;
     elseif Temp_Rand_Choice <= sum(Alternatives_Value(1:2))
        Tempchoice = 2;
     elseif Temp_Rand_Choice <= sum(Alternatives_Value(1:3))
        Tempchoice = 3;
     elseif Temp_Rand_Choice <= sum(Alternatives_Value(1:4))
        Tempchoice = 4;
     end
     %IGT_Lose_Probability
     TempLose = 0;
     if rand <= PLose(Tempchoice)
        TempLose = Lose_Proportional(Tempchoice);
     end
     %Linear operator
     Alternatives_Value(Tempchoice) =
(Memory_X*Alternatives_Value(Tempchoice))+((1-Memory_X)*(Gain_Proportional(Tempchoice)-TempLose));
     Temp_Agent_Data(Step,7) = (Memory_X*Alternatives_Value(Tempchoice));
     Temp_Agent_Data(Step,8) = ((1-Memory_X)*(Gain_Proportional(Tempchoice)-TempLose));
     %Update Alternatives_Value
     for v = 1:4
        Alternatives_Value(v) = exp(1) ^ (Alternatives_Value(v)/Temperature);
     end
     Alternatives_Value(find(Alternatives_Value<0.01)) = .01;
     Alternatives_Value = Alternatives_Value / sum(Alternatives_Value);
     Temp_Agent_Data(Step,1:6) = [Agent Tempchoice Alternatives_Value];
  end
  Data(Agent,1:2) = [Agent Temp_Group];
  for i = 1:4
     Data(Agent,i+2) = numel(find(Temp_Agent_Data(:,2)==i)) / Cards;
  end
  Block = [20:20:Cards];
  for i = 1:length(Block)
     Data(Agent,i+6) = numel(find(Temp_Agent_Data(Block(i)-(Block(1)-1):Block(i),2) == 3 |
Temp_Agent_Data(Block(i)-(Block(1)-1):Block(i),2) == 4)) / 20;
  end
  Data(Agent,[12 13 14 15]) = [numel(find(Temp_Agent_Data(:,2)==1)) numel(find(Temp_Agent_Data(:,2)==2))...
                      numel(find(Temp_Agent_Data(:,2)==3)) numel(find(Temp_Agent_Data(:,2)==4))]/Cards;
end
figure(1)
set(gcf,'position',[100 100 1000 400])
subplot(1,2,1)
hold on
Color = [.25 .25 .25];
Data_ = Data(:,7:11);
plot(1:5,nanmean(Data_), '-o','Color',Color,'MarkerFaceColor',Color,'MarkerFaceColor',Color,'LineWidth',1.5,'MarkerSize',6)
errorbar(1:5,nanmean(Data_),std(Data_)/sqrt(length(Data_)),
'.','Color',Color,'MarkerFaceColor',Color,'MarkerFaceColor',Color,'LineWidth',.5,'MarkerSize',6)
set(gca,'xlim',[.5 5.5])
```

```
set(gca,'xtick', [1:1:5])
set(gca,'ylim',[0 1])
set(gca,'ytick', [0:.2:1])
ylabel('Relative Preference Advantageus Choice','FontSize',12)
xlabel('Block','FontSize',12)
subplot(1,2,2)
hold on
Color = [.25 .25 .25];
Data_ = Data(:,12:15);
bar(1:4,nanmean(Data_),.70,'FaceColor',Color,'EdgeColor',Color,'LineWidth',1.5)
errorbar(1:4,nanmean(Data_),std(Data_)/sqrt(length(Data_)),
'.','Color',Color,'MarkerFaceColor',Color,'MarkerFaceColor',Color,'LineWidth',.5,'MarkerSize',6)
set(gca,'xlim',[.5 4.5])
set(gca,'xtick', [1:1:4])
set(gca,'xticklabel', {'A','B','C','D'})
set(gca,'ylim',[0 1])
set(gca,'ytick', [0:.2:1])
ylabel('Relative Preference','FontSize',12)
xlabel('Block','FontSize',12)
```

MATLAB Code 3 and 4 show the Iowa gambling task ABM used in the simulations and the function utilized for fitting the power function.

Code 3

Model IGTAMB fit

```
clear
clc
rng(5,'threefry')
%IGT
Cards = 100;
Gain = [100 100 50 50];
Lose =  [1250 250 250 50];
PLose = [.1 .5 .1 .5];
%Simulation
Number_Angent = 5;
%Agent
Target = [.84 .57 .22];
% Target = [.72 .44 .34];
%Data
Block = [20:20:Cards];
TempGoal = 0;
%Program
counter = 0;
while TempGoal ~= Target(1)
  counter = counter + 1;
  Data = [];
  Memory = randi([10 90],1)/100;
  Gain_Sensibility = randi([10 90],1)/100;
  Lose_Sensibility = randi([10 90],1)/100;

  for Agent = 1:Number_Angent
     %Init Agent
     Alternatives_Value = [.25 .25 .25 .25];
     Memory_X = Memory(1);
     Gain_Proportional = (Gain / max(Gain))*Gain_Sensibility(1);
     Lose_Proportional = (Lose / max(Gain))*Lose_Sensibility(1);
     Temp_Group = 1;
     for Step = 1:Cards
        %choice(Matching)
```

```
        Temp_Rand_Choice = rand;
        if Temp_Rand_Choice <= Alternatives_Value(1)
          Tempchoice = 1;
        elseif Temp_Rand_Choice <= sum(Alternatives_Value(1:2))
          Tempchoice = 2;
        elseif Temp_Rand_Choice <= sum(Alternatives_Value(1:3))
          Tempchoice = 3;
        elseif Temp_Rand_Choice <= sum(Alternatives_Value(1:4))
          Tempchoice = 4;
        end
        %IGT_Lose_Probability
        TempLose = 0;
        if rand <= PLose(Tempchoice)
          TempLose = Lose_Proportional(Tempchoice);
        end
        %Linear operator
        Alternatives_Value(Tempchoice) =
(Memory_X*Alternatives_Value(Tempchoice))+((1-Memory_X)*(Gain_Proportional(Tempchoice)-TempLose));
        Temp_Agent_Data(Step,7) = (Memory_X*Alternatives_Value(Tempchoice));
        Temp_Agent_Data(Step,8) = ((1-Memory_X)*(Gain_Proportional(Tempchoice)-TempLose));
        %Update Alternatives_Value
        Alternatives_Value(find(Alternatives_Value<0.01)) = .01;
        Alternatives_Value = Alternatives_Value / sum(Alternatives_Value);
        Temp_Agent_Data(Step,1:6) = [Agent Tempchoice Alternatives_Value];
      end
      Data(Agent,1:2) = [Agent Temp_Group];
      for i = 1:4
        Data(Agent,i+2) = numel(find(Temp_Agent_Data(:,2)==i)) / Cards;
      end
      for i = 1:length(Block)
        Data(Agent,i+6) = numel(find(Temp_Agent_Data(Block(i)-(Block(1)-1):Block(i),2) == 3 |
Temp_Agent_Data(Block(i)-(Block(1)-1):Block(i),2) == 4)) / 20;
      end
  end
  TempGoal = round(mean(Data(:,end)),2);
end
TData_1 = Data(:,7:11);
disp([1 Memory Gain_Sensibility Lose_Sensibility])
%Program
counter = 0;
while TempGoal ~= Target(2)
  counter = counter + 1;
  Data = [];
  Memory = randi([10 90],1)/100;
  Gain_Sensibility = randi([10 90],1)/100;
  Lose_Sensibility = randi([10 90],1)/100;

  for Agent = 1:(Number_Angent*2)-1
      %Init Agent
      Alternatives_Value = [.25 .25 .25 .25];
      Memory_X = Memory(1);
      Gain_Proportional = (Gain / max(Gain))*Gain_Sensibility(1);
      Lose_Proportional = (Lose / max(Gain))*Lose_Sensibility(1);
      Temp_Group = 1;
      for Step = 1:Cards
        %choice(Matching)
        Temp_Rand_Choice = rand;
        if Temp_Rand_Choice <= Alternatives_Value(1)
          Tempchoice = 1;
        elseif Temp_Rand_Choice <= sum(Alternatives_Value(1:2))
          Tempchoice = 2;
        elseif Temp_Rand_Choice <= sum(Alternatives_Value(1:3))
          Tempchoice = 3;
        elseif Temp_Rand_Choice <= sum(Alternatives_Value(1:4))
          Tempchoice = 4;
        end
        %IGT_Lose_Probability
        TempLose = 0;
        if rand <= PLose(Tempchoice)
          TempLose = Lose_Proportional(Tempchoice);
```

```
        end
        %Linear operator
        Alternatives_Value(Tempchoice) =
(Memory_X*Alternatives_Value(Tempchoice))+((1-Memory_X)*(Gain_Proportional(Tempchoice)-TempLose));
        Temp_Agent_Data(Step,7) = (Memory_X*Alternatives_Value(Tempchoice));
        Temp_Agent_Data(Step,8) = ((1-Memory_X)*(Gain_Proportional(Tempchoice)-TempLose));
        %Update Alternatives_Value
        Alternatives_Value(find(Alternatives_Value<0.01)) = .01;
        Alternatives_Value = Alternatives_Value / sum(Alternatives_Value);
        Temp_Agent_Data(Step,1:6) = [Agent Tempchoice Alternatives_Value];
      end
      Data(Agent,1:2) = [Agent Temp_Group];
      for i = 1:4
        Data(Agent,i+2) = numel(find(Temp_Agent_Data(:,2)==i)) / Cards;
      end
      for i = 1:length(Block)
        Data(Agent,i+6) = numel(find(Temp_Agent_Data(Block(i)-(Block(1)-1):Block(i),2) == 3 |
Temp_Agent_Data(Block(i)-(Block(1)-1):Block(i),2) == 4)) / 20;
      end
    end
  TempGoal = round(mean(Data(:,end)),2);
end
TData_2 = Data(:,7:11);
disp([2 Memory Gain_Sensibility Lose_Sensibility])
%Program
counter = 0;
while TempGoal ~= Target(3)
  counter = counter + 1;
  Data = [];
  Memory = randi([10 90],1)/100;
  Gain_Sensibility = randi([10 90],1)/100;
  Lose_Sensibility = randi([10 90],1)/100;

  for Agent = 1:(Number_Angent)+1
      %Init Agent
      Alternatives_Value = [.25 .25 .25 .25];
      Memory_X = Memory(1);
      Gain_Proportional = (Gain / max(Gain))*Gain_Sensibility(1);
      Lose_Proportional = (Lose / max(Gain))*Lose_Sensibility(1);
      Temp_Group = 1;
      for Step = 1:Cards
        %choice(Matching)
        Temp_Rand_Choice = rand;
        if Temp_Rand_Choice <= Alternatives_Value(1)
          Tempchoice = 1;
        elseif Temp_Rand_Choice <= sum(Alternatives_Value(1:2))
          Tempchoice = 2;
        elseif Temp_Rand_Choice <= sum(Alternatives_Value(1:3))
          Tempchoice = 3;
        elseif Temp_Rand_Choice <= sum(Alternatives_Value(1:4))
          Tempchoice = 4;
        end
        %IGT_Lose_Probability
        TempLose = 0;
        if rand <= PLose(Tempchoice)
          TempLose = Lose_Proportional(Tempchoice);
        end
        %Linear operator
        Alternatives_Value(Tempchoice) =
(Memory_X*Alternatives_Value(Tempchoice))+((1-Memory_X)*(Gain_Proportional(Tempchoice)-TempLose));
        Temp_Agent_Data(Step,7) = (Memory_X*Alternatives_Value(Tempchoice));
        Temp_Agent_Data(Step,8) = ((1-Memory_X)*(Gain_Proportional(Tempchoice)-TempLose));
        %Update Alternatives_Value
        Alternatives_Value(find(Alternatives_Value<0.01)) = .01;
        Alternatives_Value = Alternatives_Value / sum(Alternatives_Value);
        Temp_Agent_Data(Step,1:6) = [Agent Tempchoice Alternatives_Value];
      end
      Data(Agent,1:2) = [Agent Temp_Group];
      for i = 1:4
        Data(Agent,i+2) = numel(find(Temp_Agent_Data(:,2)==i)) / Cards;
```

```
      end
      for i = 1:length(Block)
         Data(Agent,i+6) = numel(find(Temp_Agent_Data(Block(i)-(Block(1)-1):Block(i),2) == 3 |
Temp_Agent_Data(Block(i)-(Block(1)-1):Block(i),2) == 4)) / 20;
      end
   end
   TempGoal = round(mean(Data(:,end)),2);
end
TData_3 = Data(:,7:11);
disp([3 Memory Gain_Sensibility Lose_Sensibility])
figure(2)
set(gcf,'position',[100 100 1000 400])
subplot(1,2,1)
hold on
Color = [.25 .25 .25];
plot(-100,-100,'-o','Color',Color,'MarkerFaceColor',Color,'MarkerFaceColor',Color,'LineWidth',.5,'MarkerSize',6)
Color = [.5 .5 .5];
plot(-100,-100,'-s','Color',Color,'MarkerFaceColor',Color,'MarkerFaceColor',Color,'LineWidth',.5,'MarkerSize',6)
Color = [.75 .75 .75];
plot(-100,-100,'-d','Color',Color,'MarkerFaceColor',Color,'MarkerFaceColor',Color,'LineWidth',.5,'MarkerSize',6)
Color = [.25 .25 .25];
plot(1:5,nanmean(TData_1), 'o','Color',Color,'MarkerFaceColor',Color,'MarkerFaceColor',Color,'LineWidth',.5,'MarkerSize',6)
errorbar(1:5,nanmean(TData_1),std(TData_1)/sqrt(length(TData_1)),
'.','Color',Color,'MarkerFaceColor',Color,'MarkerFaceColor',Color,'LineWidth',.5,'MarkerSize',6)
X = [1:1:5];
Y = nanmean(TData_1);
[fitresult, gof] = Fit_Power(X, Y);
for i = 1:500
   x(i) = fitresult.a*(i*.01)^fitresult.b;
end
plot(1.01:.01:5,x(1,101:500), '-','Color',Color,'MarkerFaceColor',Color,'MarkerFaceColor',Color,'LineWidth',1.5,'MarkerSize',6)
Text_1 = ['a=' num2str(fitresult.a,2) ' b=' num2str(fitresult.b,2)]
Color = [.5 .5 .5];
plot(1:5,nanmean(TData_2), 's','Color',Color,'MarkerFaceColor',Color,'MarkerFaceColor',Color,'LineWidth',.5,'MarkerSize',6)
errorbar(1:5,nanmean(TData_2),std(TData_2)/sqrt(length(TData_2)),
'.','Color',Color,'MarkerFaceColor',Color,'MarkerFaceColor',Color,'LineWidth',.5,'MarkerSize',6)
X = [1:1:5];
Y = nanmean(TData_2);
[fitresult, gof] = Fit_Power(X, Y);
for i = 1:500
   x(i) = fitresult.a*(i*.01)^fitresult.b;
end
plot(1.01:.01:5,x(1,101:500), '-','Color',Color,'MarkerFaceColor',Color,'MarkerFaceColor',Color,'LineWidth',1.5,'MarkerSize',6)
Text_2 = ['a=' num2str(fitresult.a,2) ' b=' num2str(fitresult.b,2)]
Color = [.75 .75 .75];
plot(1:5,nanmean(TData_3), 'd','Color',Color,'MarkerFaceColor',Color,'MarkerFaceColor',Color,'LineWidth',.5,'MarkerSize',6)
errorbar(1:5,nanmean(TData_3),std(TData_3)/sqrt(length(TData_3)),
'.','Color',Color,'MarkerFaceColor',Color,'MarkerFaceColor',Color,'LineWidth',.5,'MarkerSize',6)
X = [1:1:5];
Y = nanmean(TData_3);
[fitresult, gof] = Fit_Power(X, Y);
for i = 1:500
   x(i) = fitresult.a*(i*.01)^fitresult.b;
end
plot(1.01:.01:5,x(1,101:500), '-','Color',Color,'MarkerFaceColor',Color,'MarkerFaceColor',Color,'LineWidth',1.5,'MarkerSize',6)
Text_3 = ['a=' num2str(fitresult.a,2) ' b=' num2str(fitresult.b,2)]
title('ABM - Healthy Controls')
set(gca,'xlim',[.5 5.5])
set(gca,'xtick', [1:1:5])
set(gca,'ylim',[0 1.1])
set(gca,'ytick', [0:.2:1])
legend(['Q3: ' Text_1],['Q2: ' Text_2],['Q1: ' Text_3],'Location','northwest')
ylabel('Relative Preference Advantageus Choice','FontSize',12)
xlabel('Block','FontSize',12)
Target = [.72 .44 .34];
%Data
Block = [20:20:Cards];
TempGoal = 0;
%Program
counter = 0;
```

```
while TempGoal ~= Target(1)
  counter = counter + 1;
  Data = [];
  Memory = randi([10 90],1)/100;
  Gain_Sensibility = randi([10 90],1)/100;
  Lose_Sensibility = randi([10 90],1)/100;

  for Agent = 1:Number_Angent
     %Init Agent
     Alternatives_Value = [.25 .25 .25 .25];
     Memory_X = Memory(1);
     Gain_Proportional = (Gain / max(Gain))*Gain_Sensibility(1);
     Lose_Proportional = (Lose / max(Gain))*Lose_Sensibility(1);
     Temp_Group = 1;
     for Step = 1:Cards
        %choice(Matching)
        Temp_Rand_Choice = rand;
        if Temp_Rand_Choice <= Alternatives_Value(1)
           Tempchoice = 1;
        elseif Temp_Rand_Choice <= sum(Alternatives_Value(1:2))
           Tempchoice = 2;
        elseif Temp_Rand_Choice <= sum(Alternatives_Value(1:3))
           Tempchoice = 3;
        elseif Temp_Rand_Choice <= sum(Alternatives_Value(1:4))
           Tempchoice = 4;
        end
        %IGT_Lose_Probability
        TempLose = 0;
        if rand <= PLose(Tempchoice)
           TempLose = Lose_Proportional(Tempchoice);
        end
        %Linear operator
        Alternatives_Value(Tempchoice) =
(Memory_X*Alternatives_Value(Tempchoice))+((1-Memory_X)*(Gain_Proportional(Tempchoice)-TempLose));
        Temp_Agent_Data(Step,7) = (Memory_X*Alternatives_Value(Tempchoice));
        Temp_Agent_Data(Step,8) = ((1-Memory_X)*(Gain_Proportional(Tempchoice)-TempLose));
        %Update Alternatives_Value
        Alternatives_Value(find(Alternatives_Value<0.01)) = .01;
        Alternatives_Value = Alternatives_Value / sum(Alternatives_Value);
        Temp_Agent_Data(Step,1:6) = [Agent Tempchoice Alternatives_Value];
     end
     Data(Agent,1:2) = [Agent Temp_Group];
     for i = 1:4
        Data(Agent,i+2) = numel(find(Temp_Agent_Data(:,2)==i)) / Cards;
     end
     for i = 1:length(Block)
        Data(Agent,i+6) = numel(find(Temp_Agent_Data(Block(i)-(Block(1)-1):Block(i),2) == 3 |
Temp_Agent_Data(Block(i)-(Block(1)-1):Block(i),2) == 4)) / 20;
     end
  end
  TempGoal = round(mean(Data(:,end)),2);
end
TData_1 = Data(:,7:11);
disp([1 Memory Gain_Sensibility Lose_Sensibility])
%Program
counter = 0;
while TempGoal ~= Target(2)
  counter = counter + 1;
  Data = [];
  Memory = randi([10 90],1)/100;
  Gain_Sensibility = randi([10 90],1)/100;
  Lose_Sensibility = randi([10 90],1)/100;

  for Agent = 1:Number_Angent*2
     %Init Agent
     Alternatives_Value = [.25 .25 .25 .25];
     Memory_X = Memory(1);
     Gain_Proportional = (Gain / max(Gain))*Gain_Sensibility(1);
     Lose_Proportional = (Lose / max(Gain))*Lose_Sensibility(1);
     Temp_Group = 1;
```

```
    for Step = 1:Cards
      %choice(Matching)
      Temp_Rand_Choice = rand;
      if Temp_Rand_Choice <= Alternatives_Value(1)
        Tempchoice = 1;
      elseif Temp_Rand_Choice <= sum(Alternatives_Value(1:2))
        Tempchoice = 2;
      elseif Temp_Rand_Choice <= sum(Alternatives_Value(1:3))
        Tempchoice = 3;
      elseif Temp_Rand_Choice <= sum(Alternatives_Value(1:4))
        Tempchoice = 4;
      end
      %IGT_Lose_Probability
      TempLose = 0;
      if rand <= PLose(Tempchoice)
        TempLose = Lose_Proportional(Tempchoice);
      end
      %Linear operator
      Alternatives_Value(Tempchoice) =
(Memory_X*Alternatives_Value(Tempchoice))+((1-Memory_X)*(Gain_Proportional(Tempchoice)-TempLose));
      Temp_Agent_Data(Step,7) = (Memory_X*Alternatives_Value(Tempchoice));
      Temp_Agent_Data(Step,8) = ((1-Memory_X)*(Gain_Proportional(Tempchoice)-TempLose));
      %Update Alternatives_Value
      Alternatives_Value(find(Alternatives_Value<0.01)) = .01;
      Alternatives_Value = Alternatives_Value / sum(Alternatives_Value);
      Temp_Agent_Data(Step,1:6) = [Agent Tempchoice Alternatives_Value];
    end
    Data(Agent,1:2) = [Agent Temp_Group];
    for i = 1:4
      Data(Agent,i+2) = numel(find(Temp_Agent_Data(:,2)==i)) / Cards;
    end
    for i = 1:length(Block)
      Data(Agent,i+6) = numel(find(Temp_Agent_Data(Block(i)-(Block(1)-1):Block(i),2) == 3 |
Temp_Agent_Data(Block(i)-(Block(1)-1):Block(i),2) == 4)) / 20;
    end
  end
  TempGoal = round(mean(Data(:,end)),2);
end
TData_2 = Data(:,7:11);
disp([2 Memory Gain_Sensibility Lose_Sensibility])
%Program
counter = 0;
while TempGoal ~= Target(3)
  counter = counter + 1;
  Data = [];
  Memory = randi([10 90],1)/100;
  Gain_Sensibility = randi([10 90],1)/100;
  Lose_Sensibility = randi([10 90],1)/100;

  for Agent = 1:Number_Angent
    %Init Agent
    Alternatives_Value = [.25 .25 .25 .25];
    Memory_X = Memory(1);
    Gain_Proportional = (Gain / max(Gain))*Gain_Sensibility(1);
    Lose_Proportional = (Lose / max(Gain))*Lose_Sensibility(1);
    Temp_Group = 1;
    for Step = 1:Cards
      %choice(Matching)
      Temp_Rand_Choice = rand;
      if Temp_Rand_Choice <= Alternatives_Value(1)
        Tempchoice = 1;
      elseif Temp_Rand_Choice <= sum(Alternatives_Value(1:2))
        Tempchoice = 2;
      elseif Temp_Rand_Choice <= sum(Alternatives_Value(1:3))
        Tempchoice = 3;
      elseif Temp_Rand_Choice <= sum(Alternatives_Value(1:4))
        Tempchoice = 4;
      end
      %IGT_Lose_Probability
      TempLose = 0;
```

```
        if rand <= PLose(Tempchoice)
           TempLose = Lose_Proportional(Tempchoice);
        end
        %Linear operator
        Alternatives_Value(Tempchoice) =
(Memory_X*Alternatives_Value(Tempchoice))+((1-Memory_X)*(Gain_Proportional(Tempchoice)-TempLose));
        Temp_Agent_Data(Step,7) = (Memory_X*Alternatives_Value(Tempchoice));
        Temp_Agent_Data(Step,8) = ((1-Memory_X)*(Gain_Proportional(Tempchoice)-TempLose));
        %Update Alternatives_Value
        Alternatives_Value(find(Alternatives_Value<0.01)) = .01;
        Alternatives_Value = Alternatives_Value / sum(Alternatives_Value);
        Temp_Agent_Data(Step,1:6) = [Agent Tempchoice Alternatives_Value];
     end
     Data(Agent,1:2) = [Agent Temp_Group];
     for i = 1:4
        Data(Agent,i+2) = numel(find(Temp_Agent_Data(:,2)==i)) / Cards;
     end
     for i = 1:length(Block)
        Data(Agent,i+6) = numel(find(Temp_Agent_Data(Block(i)-(Block(1)-1):Block(i),2) == 3 |
Temp_Agent_Data(Block(i)-(Block(1)-1):Block(i),2) == 4)) / 20;
     end
  end
  TempGoal = round(mean(Data(:,end)),2);
end
TData_3 = Data(:,7:11);
disp([3 Memory Gain_Sensibility Lose_Sensibility])
subplot(1,2,2)
hold on
Color = [.25 .25 .25];
plot(-100,-100,'-o','Color',Color,'MarkerFaceColor',Color,'MarkerFaceColor',Color,'LineWidth',.5,'MarkerSize',6)
Color = [.5 .5 .5];
plot(-100,-100,'-s','Color',Color,'MarkerFaceColor',Color,'MarkerFaceColor',Color,'LineWidth',.5,'MarkerSize',6)
Color = [.75 .75 .75];
plot(-100,-100,'-d','Color',Color,'MarkerFaceColor',Color,'MarkerFaceColor',Color,'LineWidth',.5,'MarkerSize',6)
Color = [.25 .25 .25];
plot(1:5,nanmean(TData_1), 'o','Color',Color,'MarkerFaceColor',Color,'MarkerFaceColor',Color,'LineWidth',.5,'MarkerSize',6)
errorbar(1:5,nanmean(TData_1),std(TData_1)/sqrt(length(TData_1)),
'.','Color',Color,'MarkerFaceColor',Color,'MarkerFaceColor',Color,'LineWidth',.5,'MarkerSize',6)
X = [1:1:5];
Y = nanmean(TData_1);
[fitresult, gof] = Fit_Power(X, Y);
for i = 1:500
  x(i) = fitresult.a*(i*.01)^fitresult.b;
end
plot(1.01:.01:5,x(1,101:500), '-','Color',Color,'MarkerFaceColor',Color,'MarkerFaceColor',Color,'LineWidth',1.5,'MarkerSize',6)
Text_1 = ['a=' num2str(fitresult.a,2) ' b=' num2str(fitresult.b,2)]
Color = [.5 .5 .5];
plot(1:5,nanmean(TData_2), 's','Color',Color,'MarkerFaceColor',Color,'MarkerFaceColor',Color,'LineWidth',.5,'MarkerSize',6)
errorbar(1:5,nanmean(TData_2),std(TData_2)/sqrt(length(TData_2)),
'.','Color',Color,'MarkerFaceColor',Color,'MarkerFaceColor',Color,'LineWidth',.5,'MarkerSize',6)
X = [1:1:5];
Y = nanmean(TData_2);
[fitresult, gof] = Fit_Power(X, Y);
for i = 1:500
  x(i) = fitresult.a*(i*.01)^fitresult.b;
end
plot(1.01:.01:5,x(1,101:500), '-','Color',Color,'MarkerFaceColor',Color,'MarkerFaceColor',Color,'LineWidth',1.5,'MarkerSize',6)
Text_2 = ['a=' num2str(fitresult.a,2) ' b=' num2str(fitresult.b,2)]
Color = [.75 .75 .75];
plot(1:5,nanmean(TData_3), 'd','Color',Color,'MarkerFaceColor',Color,'MarkerFaceColor',Color,'LineWidth',.5,'MarkerSize',6)
errorbar(1:5,nanmean(TData_3),std(TData_3)/sqrt(length(TData_3)),
'.','Color',Color,'MarkerFaceColor',Color,'MarkerFaceColor',Color,'LineWidth',.5,'MarkerSize',6)
X = [1:1:5];
Y = nanmean(TData_3);
[fitresult, gof] = Fit_Power(X, Y);
for i = 1:500
  x(i) = fitresult.a*(i*.01)^fitresult.b;
end
plot(1.01:.01:5,x(1,101:500), '-','Color',Color,'MarkerFaceColor',Color,'MarkerFaceColor',Color,'LineWidth',1.5,'MarkerSize',6)
Text_3 = ['a=' num2str(fitresult.a,2) ' b=' num2str(fitresult.b,2)]
```

```
title('ABM - Substance Abusers')
set(gca,'xlim',[.5 5.5])
set(gca,'xtick', [1:1:5])
set(gca,'ylim',[0 1.1])
set(gca,'ytick', [0:.2:1])
legend(['Q3: ' Text_1],['Q2: ' Text_2],['Q1: ' Text_3],'Location','northwest')
% ylabel('Relative Preference Advantageus Choice','FontSize',12)
xlabel('Block','FontSize',12)
```

Code 4

Power Function

```
function [fitresult, gof] = Fit_Power(X, Y)
[xData, yData] = prepareCurveData( X, Y );
ft = fittype( 'power1' );
opts = fitoptions( 'Method', 'NonlinearLeastSquares' );
opts.Display = 'Off';
opts.StartPoint = [0 0];
[fitresult, gof] = fit( xData, yData, ft, opts );
```

In order to evaluate and explore alternative adjustment methods, the Nelder-Mead algorithm along with quadratic error was employed to estimate the memory values, gain sensitivity, and loss sensitivity of the agents. Our objective was to achieve the best fit between the relative preferences of participants across 5 blocks and within those blocks, utilizing Python and the scipy.optimize and minimize libraries. Parameters were refined to minimize error within a maximum of 1000 iterations (Code 5).

Code 5

Model Adjustment using Nelder-Mead

```
import numpy as np
from scipy.optimize import minimize
import pandas as pd

np.random.default_rng(seed=5)

import numpy as np
from scipy.optimize import minimize
import pandas as pd

def optimize_AIGT(y_data, options={'maxiter': 100}, tol=0.001):
    best_parameters = None
    best_result = float('inf')

    def AIGT(M, G, L):

        np.random.default_rng(seed=5)

        Cards = 100
```

```
        Gain = np.array([100, 100, 50, 50])
        Lose = np.array([1250, 250, 250, 50])
        PLose = np.array([0.1, 0.5, 0.1, 0.5])

        Memory = M
        Gain_Sensibility = G
        Lose_Sensibility = L

        Agent = 1

        Alternatives_Value = np.array([0.25, 0.25, 0.25, 0.25])
        Memory_X = Memory
        Gain_Proportional = (Gain / np.max(Gain)) * Gain_Sensibility
        Lose_Proportional = (Lose / np.max(Gain)) * Lose_Sensibility

        Temp_Agent_Data = np.zeros((Cards, 8))

        for Step in range(Cards):
            Temp_Rand_Choice = np.random.rand()
            if Temp_Rand_Choice <= Alternatives_Value[0]:
                Tempchoice = 1
            elif Temp_Rand_Choice <= np.sum(Alternatives_Value[:2]):
                Tempchoice = 2
            elif Temp_Rand_Choice <= np.sum(Alternatives_Value[:3]):
                Tempchoice = 3
            elif Temp_Rand_Choice <= np.sum(Alternatives_Value[:4]):
                Tempchoice = 4

            TempLose = 0
            if np.random.rand() <= PLose[Tempchoice - 1]:
                TempLose = Lose_Proportional[Tempchoice - 1]

            Alternatives_Value[Tempchoice - 1] = (Memory_X * Alternatives_Value[Tempchoice - 1]) + ((1 - Memory_X) *
(Gain_Proportional[Tempchoice - 1] - TempLose))
            Temp_Agent_Data[Step, 6] = (Memory_X * Alternatives_Value[Tempchoice - 1])
            Temp_Agent_Data[Step, 7] = ((1 - Memory_X) * (Gain_Proportional[Tempchoice - 1] - TempLose))

            Alternatives_Value[Alternatives_Value < 0.01] = 0.01
            Alternatives_Value /= np.sum(Alternatives_Value)
            Temp_Agent_Data[Step, :6] = [Agent, Tempchoice] + list(Alternatives_Value)

        Block = np.arange(20, Cards + 1, 20)
        AData = np.zeros(len(Block))
        for i in range(len(Block)):
            AData[i] = np.count_nonzero((Temp_Agent_Data[Block[i] - (Block[0] - 1):Block[i], 1] == 3) | (Temp_Agent_Data[Block[i] - (Block[0]
- 1):Block[i], 1] == 4)) / 20

        return AData

    def error_quadratic_mean(parameters, x, y):
        M, G, L = parameters
        y_pred = AIGT(M, G, L)
        error = np.mean((y - y_pred) ** 2)
        return error

    x_data = np.array([1, 2, 3, 4, 5])

    initial_guess = [0.5, 0.5, 0.5]
    iteration_results = []

    def callback(iteration_parameters):
        M, G, L = iteration_parameters
        y_pred = AIGT(M, G, L)
        error = np.mean((y_data - y_pred) ** 2)
        iteration_results.append({'Iteration': len(iteration_results) + 1, 'Error': error, 'Parameters': iteration_parameters, 'y_pred': y_pred})

    result = minimize(error_quadratic_mean, initial_guess, args=(x_data, y_data), method='Nelder-Mead', options=options, tol=tol,
callback=callback)
    DataA = pd.DataFrame(iteration_results)
    predicted = DataA.iloc[DataA['Error'].idxmin()]
```

```
    return predicted

# Example usage:
y_data = np.array([0.40, 0.60, 0.40, 0.80, 0.85])
predicted_result = optimize_AIGT(y_data, options={'maxiter': 100}, tol=0.001)
print(predicted_result)
```

Figure 1 presents the participants' results across the 5 blocks, while Figure 2 illustrates the results of agents using the Nelder-Mead method. Notably, the model predictions, particularly for the SUD group, showed qualitative improvement compared to the predictions depicted in Figure 3, where the adjustment method described in the manuscript was employed.

Figure 1

Participants Mean relative preference for advantageous choices for groups, subgroups, and blocks.

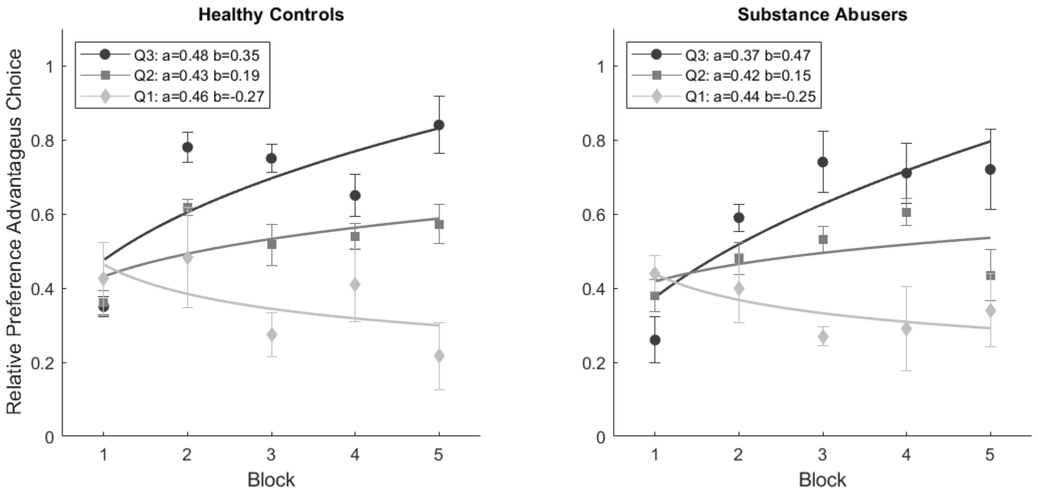

Figure 2

ABM-Nelder-Mead *Mean relative preference for advantageous choices for groups, subgroups, and blocks.*

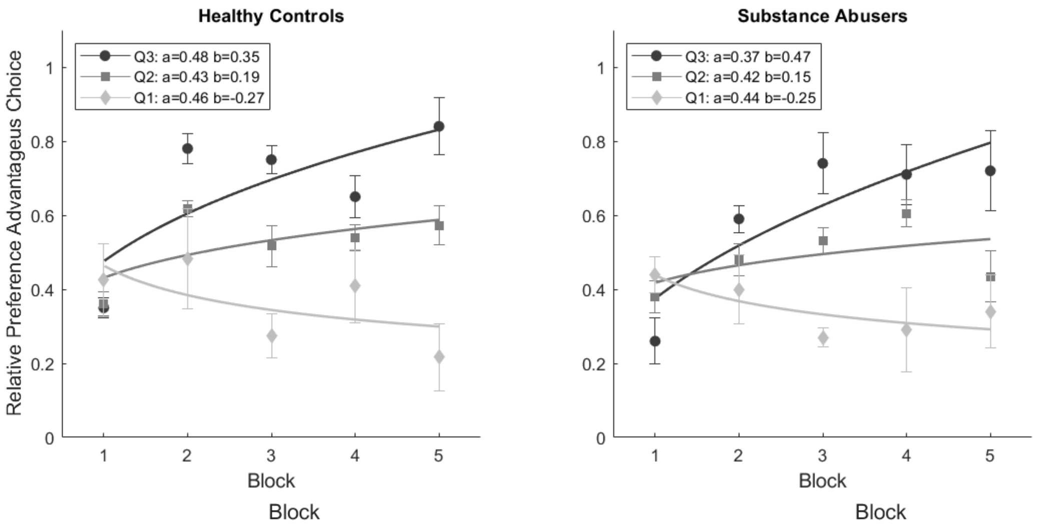

Figure 3

ABM Mean relative preference for advantageous choices for groups, subgroups, and blocks.

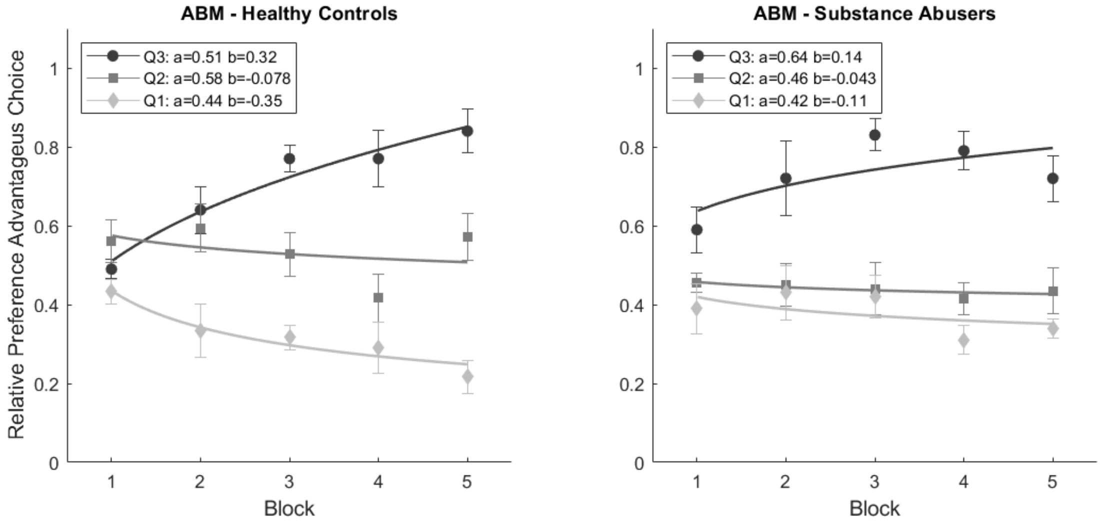

To assess the relationship between the Mean Relative Preference for Advantageous Choices of participants and the Agent-Based Model using parameters recovered with the Nelder-Mead method, as well as the relationship between the preference for advantageous choices in the

simulation of the adjusted Agent-Based Model and its replicas with the recovered parameters, correlations were employed. In Figure 4A, the correlation between participants' preference and the model is depicted. This correlation (r = 0.96, p, 0.001) is higher compared to the correlation (*r* = 0.79, *p* < 0.001) of the data using the fitting method described in the manuscript (Figure 6A). However, the parameters fail (r = .038, p = 0.64) to replicate the data generated by the model again (Figure 4B). On the other hand, the replicas generated with the described fitting method (Figure 5B) do show a statistically significant correlation (r = 0.89, p ,0.001). This is due to the characteristics of the model and the task. Given that agents learn to choose based on their experience with the task, which largely depends on the probability of punishments, it was possible that initial values of memory, gain sensitivity, and loss sensitivity of 0.5 could show randomly a preference similar to that of participants. In this sense, adjusting using the Nelder-Mead method could make small adjustments to parameter values (e.g., 0.001) without exploring large ranges of variation (e.g., 0.1).

Figure 4

Nelder-Mead Correlation Relative Preference for Advantageous Choices .

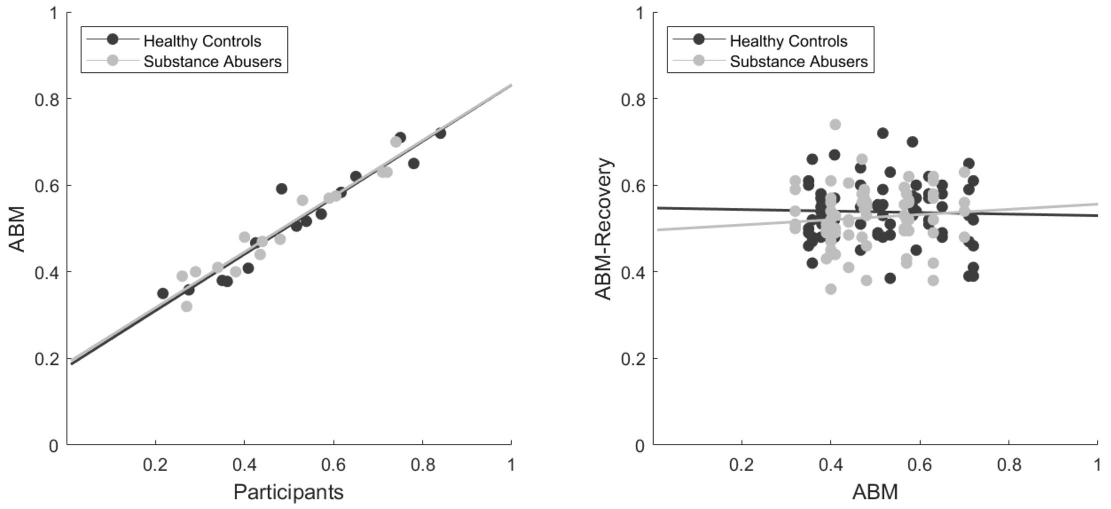

Figure 5

Correlation Relative Preference for Advantageous Choices .

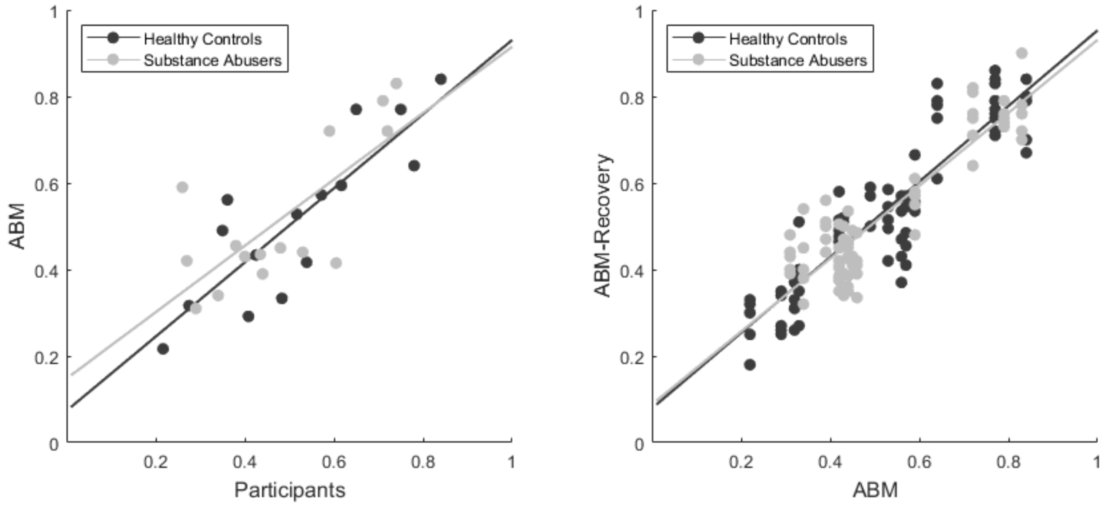

Figures 6A and 6B display the recovered values using Nelder-Mead. It can be appreciated that the parameter values do not differ significantly from the initial parameters. Similar to the fitting described in the manuscript, agents from group Q3 tended to exhibit higher sensitivity to rewards and lower sensitivity to gains compared to groups Q1. However, this fitting method does not capture the effects of memory equally well for groups Q2 (see table 1)

Figure 6

Nelder-Mead ABM Simulations Estimated Parameter Values .

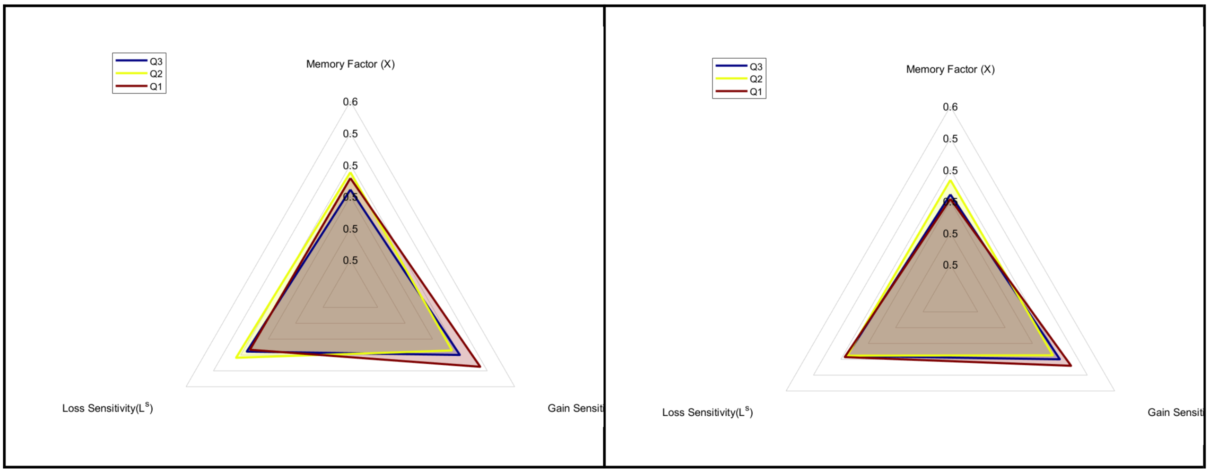

Table 1

Nelder-Mead ABM Simulations Estimated Parameter Values .

	**Control**			**Sud**	
**M**	**GS**	**LS**	**M**	**GS**	**LS**
Q1	0.494	0.510	0.506	0.494	0.510
Q2	0.505	0.504	0.514	0.503	0.505
Q3	0.502	0.525	0.503	0.491	0.518
